# Evaluating the Efficacy of ChatGPT in Navigating the Spanish Medical Residency Entrance Examination (MIR): Promising Horizons for AI in Clinical Medicine

**DOI:** 10.3390/clinpract13060130

**Published:** 2023-11-20

**Authors:** Francisco Guillen-Grima, Sara Guillen-Aguinaga, Laura Guillen-Aguinaga, Rosa Alas-Brun, Luc Onambele, Wilfrido Ortega, Rocio Montejo, Enrique Aguinaga-Ontoso, Paul Barach, Ines Aguinaga-Ontoso

**Affiliations:** 1 Department of Health Sciences, Public University of Navarra, 31008 Pamplona, Spain; sguillen.4@alumni.unav.es (S.G.-A.); guillen.124514@e.unavarra.es (L.G.-A.); rosamaria.alas@unavarra.es (R.A.-B.); 2 Healthcare Research Institute of Navarra (IdiSNA), 31008 Pamplona, Spain; 3 Department of Preventive Medicine, Clinica Universidad de Navarra, 31008 Pamplona, Spain; 4 CIBER in Epidemiology and Public Health (CIBERESP), Institute of Health Carlos III, 46980 Madrid, Spain; 5 Department of Nursing, Kystad Helse-og Velferdssenter, 7026 Trondheim, Norway; 6 School of Health Sciences, Catholic University of Central Africa, Yaoundé 1100, Cameroon; onambele.luc@ess-ucac.org; 7 Department of Surgery, Medical and Social Sciences, University of Alcala de Henares, 28871 Alcalá de Henares, Spain; wilfrido.ortega@edu.uah.es; 8 Department of Obstetrics and Gynecology, Institute of Clinical Sciences, University of Gothenburg, 413 46 Gothenburg, Sweden; rocio.montejo.rodriguez@gu.se; 9 Department of Obstetrics and Gynecology, Sahlgrenska University Hospital, 413 46 Gothenburg, Sweden; 10 Department of Sociosanitary Sciences, University of Murcia, 30100 Murcia, Spain; aguinaga@um.es; 11 Jefferson College of Population Health, Philadelphia, PA 19107, USA; paul.barach@jefferson.edu; 12 School of Medicine, Thomas Jefferson University, Philadelphia, PA 19107, USA; 13 Interdisciplinary Research Institute for Health Law and Science, Sigmund Freud University, 1020 Vienna, Austria; 14 Department of Surgery, Imperial College, London SW7 2AZ, UK

**Keywords:** machine learning, artificial intelligence, ChatGPT, GPT-3.5, GPT-4, medical education, quality of care, patient safety, image, large language model

## Abstract

The rapid progress in artificial intelligence, machine learning, and natural language processing has led to increasingly sophisticated large language models (LLMs) for use in healthcare. This study assesses the performance of two LLMs, the GPT-3.5 and GPT-4 models, in passing the MIR medical examination for access to medical specialist training in Spain. Our objectives included gauging the model’s overall performance, analyzing discrepancies across different medical specialties, discerning between theoretical and practical questions, estimating error proportions, and assessing the hypothetical severity of errors committed by a physician. Material and methods: We studied the 2022 Spanish MIR examination results after excluding those questions requiring image evaluations or having acknowledged errors. The remaining 182 questions were presented to the LLM GPT-4 and GPT-3.5 in Spanish and English. Logistic regression models analyzed the relationships between question length, sequence, and performance. We also analyzed the 23 questions with images, using GPT-4’s new image analysis capability. Results: GPT-4 outperformed GPT-3.5, scoring 86.81% in Spanish (*p* < 0.001). English translations had a slightly enhanced performance. GPT-4 scored 26.1% of the questions with images in English. The results were worse when the questions were in Spanish, 13.0%, although the differences were not statistically significant (*p* = 0.250). Among medical specialties, GPT-4 achieved a 100% correct response rate in several areas, and the Pharmacology, Critical Care, and Infectious Diseases specialties showed lower performance. The error analysis revealed that while a 13.2% error rate existed, the gravest categories, such as “error requiring intervention to sustain life” and “error resulting in death”, had a 0% rate. Conclusions: GPT-4 performs robustly on the Spanish MIR examination, with varying capabilities to discriminate knowledge across specialties. While the model’s high success rate is commendable, understanding the error severity is critical, especially when considering AI’s potential role in real-world medical practice and its implications for patient safety.

## 1. Introduction

Artificial Intelligence (AI) is revolutionizing patient care by enhancing diagnostic accuracy, personalizing treatment plans, and in streamlining workflows, thus augmenting the capabilities of healthcare providers and potentially improving health outcomes. Integrating AI in clinical medicine is depicted as a transformative force, fundamentally enhancing various facets of healthcare. The emerging literature accentuates AI’s role in augmenting diagnostic precision, tailoring treatment plans, and refining healthcare delivery. Notably, AI’s inclusion into medical practice has been reported to lead to more efficient healthcare delivery, cost reductions, and bolstering treatment outcomes [1].

Incorporating AI in clinical medicine signifies a paradigm shift, with the potential to profoundly reshape patient care and medical research. AI, with its advanced algorithms and data-processing capabilities, promises to revolutionize healthcare by enhancing diagnostic accuracy, facilitating the development of personalized treatment plans, and assisting clinicians in complex decision-making processes [2]. The transition to AI-driven medicine is not merely about task automation but a potential leap toward sophisticated technologies that augment patient care across various healthcare settings [3]. This shift is characterized by improved efficiency in healthcare delivery, which has notable implications for reducing medical costs. AI’s impact on healthcare is multifaceted, with improvements in treatment outcomes stemming from its ability to integrate and analyze vast arrays of medical data leading to more informed and precise clinical decisions. Moreover, AI’s role in medicine encompasses various applications, ranging from managing patient histories to automating diagnostic tools such as reading MRI and CT scans. AI has been shown to contribute to better management of stocks and sale records in pharmacy [4].

In 2023, 172 devices equipped with AI received FDA approval for marketing in the United States. Of those, 79% were in Radiology [5]. AI is increasingly being used in image-intensive fields like ophthalmology, where AI’s potential for advancing diagnosis and treatment efficacy is being realized [6,7], and in endoscopy and colonoscopy, where high-accuracy AI image recognition can be used for disease identification, treatment planning, and improved bowel preparation for colonoscopy [8,9]. Artificial AI is also widespread in ultrasound imaging in obstetrics, focusing primarily on fetal biometry [10] and pediatrics [11]. AI is growing in enhancing the interpretation of medical images in neurology for conditions like epilepsy, stroke, multiple sclerosis, Alzheimer’s, and Parkinson’s disease [12,13,14]. AI has been used in improving the radiological diagnosis of colorectal cancer. It has also been used as an aid for the diagnosis of histological biopsy slides of prostate and color cancer [15,16]. AI can be used for quality assurance of images in radiology [17].

AI is being increasingly applied in gastroenterology and hepatology to improve outcomes. AI aids liver disease management by providing tools for detecting early diseases in blood donors, characterizing disease severity, and quantifying treatment responses [18]. Finally, AI has contributed to liver transplantation by improving the quantification of steatosis in donors’ livers, potentially leading to better predictions of transplantation outcomes [19]. AI can enhance the postoperative quality of life prediction in bariatric surgery cases [20]. AI is also advancing the diagnosis of celiac disease, with various studies employing algorithms for quick and accurate tests and identifying biomarkers [21].

AI is revolutionizing cardiology by enhancing cardiovascular disease prediction, diagnosis, and treatment, with some models outperforming cardiologists in identifying cardiac rhythms [22]. The focus has been on refining diagnostic and predictive tools using hospital data, ECGs, and echocardiograms. AI’s capabilities include predicting hypertension from health records, diagnosing atrial and ventricular fibrillation, and increasing the precision of ventricular volume and function measurements from MRI scans [23,24,25].

Despite the significant inroads made by AI in clinical medicine, the nuanced nature of clinical decision making in this field demands more than just pattern recognition and predictive analytics. Bridging the gap between data-driven forecasts and the multifaceted reasoning required in medical practice is a considerable challenge. It requires AI to interpret vast amounts of data and understand and generate human-like texts to assist decision-making processes [26,27]. The application of AI in healthcare goes beyond the raw analysis of numerical data, venturing into the realm of natural language processing (NLP) to contextualize and streamline the complexities of medical data interpretation [28,29]. This intersection between advanced language models and clinical expertise heralds a new frontier where the synergy between AI and human clinicians could lead to unprecedented improvements in patient care and medical research [30].

The advances in AI, especially in natural language processing, have ushered in new opportunities. Since its appearance on 30 November 2021, ChatGPT has resulted (as of 23 July 2023) in more than 1096 scientific articles indexed on Scopus, of which 26% are related to health sciences [31]. A search performed by the authors in Pubmed (as of 14 November) resulted in 1719 articles. ChatGPT refers to the conversational interface built by OpenAI’s Generative Pre-Trained Transformer (GPT) large language models (LLM), designed to engage in natural language interactions such as GPT-3.5 and GPT-4. While the term “ChatGPT” is used generically in this paper to denote the conversational capabilities of these architectures, we will specify either GPT-3.5 or GPT-4 when discussing attributes or findings related to a particular LLM version. The free version uses the GPT-3.5 model. The premium version that employs GPT-4 is recommended [32]. GPT-4 was developed by self-training to forecast subsequent sentence words by intermittently concealing input words. ChatGPT models have shown diverse applicability, but their performance in specialized medical examinations remains an area of deep interest. While predicting upcoming words is relevant for language creation, it is not directly applicable to diverse health datasets like physiological waveforms due to the complexity and depth of understanding needed to decipher and infer actions in medical decision-making [33]. Generative AI and large language models are sparking new conversations about the future of healthcare, hinting at a landscape where AI tools like ChatGPT could significantly enhance healthcare education, research, and the practical aspects of clinical practice [34].

ChatGPT has shown promise in various medical fields, including allergology, dermatology, and radiology [32,35,36], and in a pool of questions formulated by physicians of seventeen specialties [37]. Nevertheless, the performance has not been consistently good. However, GPT-4 failed to pass the American College of Gastroenterology Self-Assessment examination [38].

ChatGPT has been evaluated with real questions that patients ask physicians [39]. It has been tested in the Medical Licensing Examination of the United States [40,41], the German State Examination in Medicine [42], the China National Medical Licensing Examination, and the China National Entrance Examination for Postgraduate Clinical Medicine Comprehensive Ability [43], Taiwan’s Examination for Medical Doctors [44], and the Japanese Medical Licensing Examination [45]. A meta-analysis of examinations from several medical specialties and several countries found ChatGPT’s overall performance of 61.1% (95% CI 56.1–66.0%), but this was made with GPT 3.5 [46].

The primary aim of this study is to critically assess the proficiency of the LLM GPT-3.5 and GPT-4 models in passing the MIR medical examination. The MIR exam serves as the gateway to medical specialist training in Spain. This rigorous test evaluates candidates’ knowledge using a multiple-choice questionnaire, which primarily aims to determine a priority competency score for selecting a specialty and hospital. The efficacy of LLM in navigating specialized medical examinations, such as the MIR (“Médico Interno Residente”) medical examination in Spain, is unknown.

We endeavor to gauge the overall performance, delve into potential performance variations across distinct medical specialties, and distinguish the LLM capabilities in handling theoretical versus practical questions. An integral part of this investigation also involves estimating the proportion of errors in the LLM responses. Additionally, we are keen to discern the potential repercussions of such errors, imagining a scenario where a practicing physician might have committed them.

Several hypotheses underpin this research. Firstly, we anticipate that the models might exhibit enhanced aptitude in resolving theoretical questions compared to practical ones. Furthermore, we predict that the more recent GPT-4 model will surpass GPT-3.5 in performance. Differences in performance across various medical specialties are also expected. Intriguingly, we hypothesize that the sequence of the presented questions might influence the models’ outputs, possibly attributed to model “fatigue”. Our research also considers linguistic nuances, postulating that the models’ performance may be more optimized for English questions than ones in Spanish. Lastly, the length of the questions might significantly influence the quality and accuracy of the LLM’s responses.

## 2. Materials and Methods

### 2.1. Context—The Graduate Medication Education System in Spain

In Spain, there are 47 registered medical specialties. A medical specialty typically necessitates spending 4 to 5 years as a Medical Intern (MIR) at a hospital or an accredited health center [47] In 2023, 8550 specialty positions were made available across Spain, most of which 8316 were situated in public hospitals and health centers, while the remaining 234 positions were offered in private health systems.

The MIR exam was taken in 2023 by 11,578 doctors, comprising 8685 Spanish and 2893 foreign practitioners. Interestingly, foreigners were granted 16.37% of the MIR positions in 2023. Specifically, 1378 doctors from non-European Union nations secured a job through the MIR exam during the same year [48].

### 2.2. The Spanish Medical Intern Examination-MIR Exam

The MIR exam comprises 200 multiple-choice questions with four potential answers—only one of which is correct. In addition, there are ten reserve questions to address issues related to question formulation or typographical errors, bringing the total to 210 questions. The exam does not adhere to a specified official syllabus; it may include questions on any aspect of medicine and typically draws from topics found in commonly used medical textbooks.

The exam lasted 4 h and 30 min and is administered in venues rented by the Ministry of Health throughout Spain. The score from the exam contributes to 90% of the final grade for specialty placement, with the remaining 10% coming from the candidate’s academic record. Based on these grades, candidates are ranked, subsequently determining the selection of specialty training spots.

**Figure 1:**
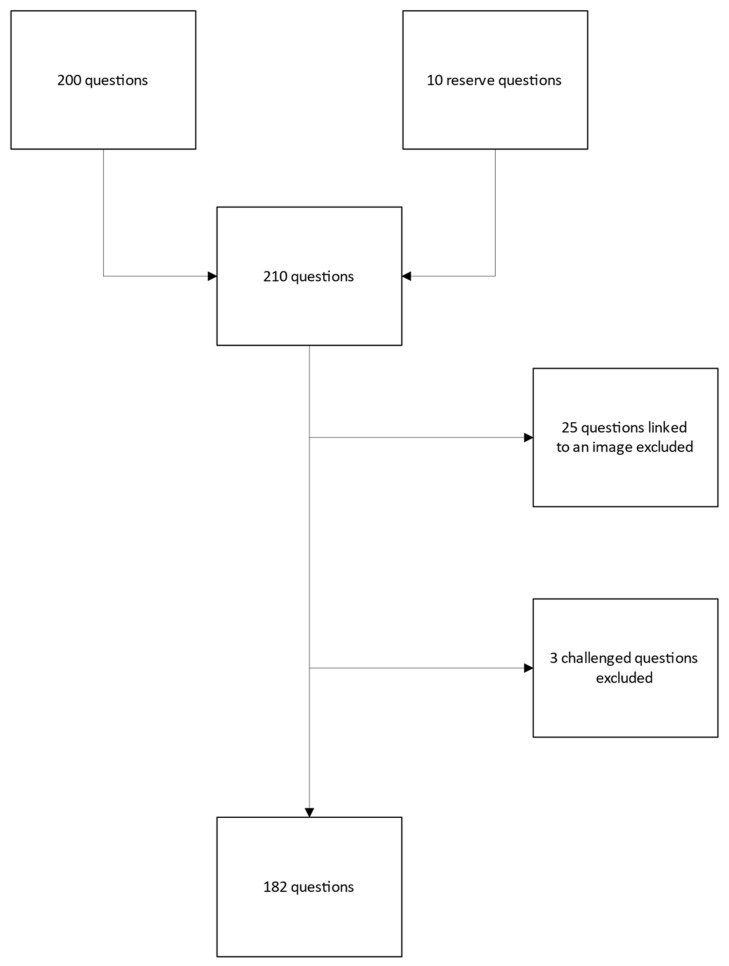
Study flow diagram: Question selection process and exclusion criteria.

We obtained the 2022 Spanish Medical Residency Entrance Exam (MIR) questions from the Spanish Ministry of Health website (https://fse.mscbs.gob.es/fseweb/view/public/datosanteriores/cuadernosExamen/busquedaConvocatoria.xhtml (accessed on 5 November 2023)). The MIR examination has a master version (Type 0) and four examination types (1 to 4) [49]. All the versions of the examinations have the same questions, but the order of questions and answers may differ. From the five available examinations on the Ministry of Health website, we chose type 0. We eliminated all questions that required an evaluation of images, like an MRI or an ECG. We also eliminated those questions challenged by the doctors who sat for the exam and were accepted by the Ministry of Health, generally because of ambiguity in the wording or because there were several positive answers. We also included the reserve questions. This left us with 182 questions. Only those questions that required reading of a text were included in the comparative analysis. The flow diagram is presented in Figure 1.

We prompted the questions to GPT-4 (August 2023 version) and GPT-3.5 [50]. We prompted the questions in Spanish and English. We used the prompt: “Please answer the following multiple-choice questions. Note: These questions are from the Medical Intern Resident (MIR) exam taken by doctors in Spain. The answers to the questions are universal; however, some questions may have nuances specific to Spain, especially in areas related to the vaccination calendar, list of notifiable diseases, legal aspects, and organization of health services. Answer with only the question number and the number of the correct answer option”. We prompted the questions in blocks of 10 questions.

The questions were divided into two groups: theoretical and practical. Questions were also classified according to their specialty in the following groups: Cardiology, Dermatology, Endocrinology, Epidemiology, Ethics, Family and Community Medicine, Gastroenterology, Genetics, Geriatrics, Hematology, Immunology, Infectious Diseases, Legal and Forensic Medicine, Maxillofacial Surgery, Nephrology, Obstetrics and Gynecology, Orthopedic and Traumatology Surgery, Otorhinolaryngology, Pathology, Pharmacology, Physiopathology, and Plastic Surgery.

The English translation of the questions and the correct answers are presented in Supplements Tables S1 and S2. We computed the number of words and characters for each question in Spanish using Microsoft Word 365 (Microsoft^®^ Word 365 MSO version 2307). We also calculated the number of tokens using the AI Tokenizer (https://platform.openai.com/tokenizer (accessed on 5 November 2023)).

To assess the existence of nonrandom error, we submitted the test three times to GPT-4, two times with the questions in the original order and another time with the questions in a random order, and comparisons were made between the different versions.

We assessed the potential risks to the patient in a hypothetical scenario in which the failure to adequately answer the question would have occurred in real life. We evaluated the potential risks for patients of failing key questions using the National Coordinating Council for Medication Error Reporting and Prevention (NCC MERP) classification system [51]. This classification has four categories of error: no error, error-no harm, error-harm, and error-death. It also has the following subcategories:

- Category A. Circumstances or events that have the capacity to cause error.
- Category B. An error occurred, but the error did not reach the patient.
- Category C. An error occurred that reached the patient but did not cause the patient harm.
- Category D. An error occurred that reached the patient and required monitoring to confirm that it resulted in no harm to the patient or required intervention to preclude harm.
- Category E. An error that may have contributed to or resulted in temporary harm to the patient and required intervention.
- Category F. An error occurred that may have contributed to or resulted in temporary harm to the patient and required initial or prolonged hospitalization.
- Category G. An error occurred that may have contributed to or resulted in permanent patient harm.
- Category H. An error occurred that required intervention necessary to sustain life.
- Category I. An error occurred that may have contributed to or resulted in the patient’s death [51].

The questions GPT-4 failed in the MIR examination, indicating the correct answer, wrong answer, and the error classification are presented in Supplement Table S3.

The questionnaire in Spanish was presented twice to the GPT-4 in its original sequence, termed “Original Question Sequence”, to discern any patterns in the responses. These attempts were called the “First Attempt” and “Second Attempt”, respectively. Subsequently, the questions were reordered randomly to form the “Random Question Sequence”. GPT-4’s responses to this shuffled set were then evaluated in two distinct manners: once in the randomized sequence itself (“Evaluated in Random Order”) and once after sorting the outcomes according to the original MIR examination sequence (“Evaluated in Original Sequence”). The consistency and predictability of the answers across these scenarios were quantified using the Runs Test, with various metrics such as the Test Value, Number of Runs, Z-value, and Asymptotic Significance being recorded for a comprehensive comparison.

### 2.3. Image Processing

GPT-4 allows for an experimental image capability that included uploading photos. This experimental feature was deployed in Spain in October 2023. Using this feature, all the questions with photos of the MIR examination were uploaded except for one question that was excluded because it had been challenged and removed by the Ministry of Health. That accrued to twenty-four images. This experimental feature was run under the GPT-4 September version [52]. The questions’ images and English translation are available in Supplement Table S4. The images in the text were the X-ray of thorax (3), microscopic slides (3), photos (3), electrocardiogram (2), abdominal CT with intravenous contrast (1), abdominal ultrasound (1), abdominopelvic CT (1), brain MRI (1), cervicofacial CT (1), eye fundus examination (1), genealogy (1), hysterosalpingography (1), laryngoscopy image (1), macro and microscopic slide(1), PET/CT (1), pyrophosphate scintigraphy (1), and respiratory polygraphy (1). It has been proven that GPT can solve issues involving images based on text without having access to the images [53]. For this reason, the questions were calculated using images and without loading the images.

### 2.4. Statistical Analysis

We computed confidence intervals of proportions and Cohen’s Kappa, a statistic used to measure inter-rater reliability for categorical items. The calculations were made using OpenEpi v. 3.01 [54]. All the remaining analyses were performed using IBM SPSS version 26. We calculated the Wald–Wolfowitz Runs Test, a non-parametric statistical test that checks the randomness of a data sequence to see if there was an aggregation on the error. We compared the results using the McNemar Test between the GPT-3.5 and GPT-4 versions and the Spanish and English versions of the questionnaire. We computed Cohen’s Kappa to study the concordance of the results between the first and second attempts to submit the examinations to GPT-4. We also calculated the Chi-square test, as well as adjusted standardized residuals.

We used developed logistic multivariate regression models to see if the number of words, characters, and tokens were related to failing a question. We also used Polynomial Logistic Regression, introducing square terms in the models. As polynomial terms, especially when squared or cubed, can lead to multi-collinearity problems, we centered the variables (subtracting their mean) before squaring.

We also performed logistic regressions using lags (lag1 and lag2) of the correct question with the results of both the Spanish version of the questionnaire and the random version with GPT-4.

## 3. Results

The LLM GPT-4 proportion of correct answers in Spanish was 86.81%, higher than that of GPT-3.5 at 63.18%. (*p* < 0.001) (Table 1) The same happened with the English translation of the questionnaire. When the English questionnaire was used, the responses improved slightly by 1.10% with GPT-4 and 3.30% with GPT-3.5, but these differences were not statistically significant.

From here on, we will only report on the GPT-4 experience with questions in Spanish. There was no difference in the results between theoretical and practical questions in the examination (Table 2).

**Figure 2:**
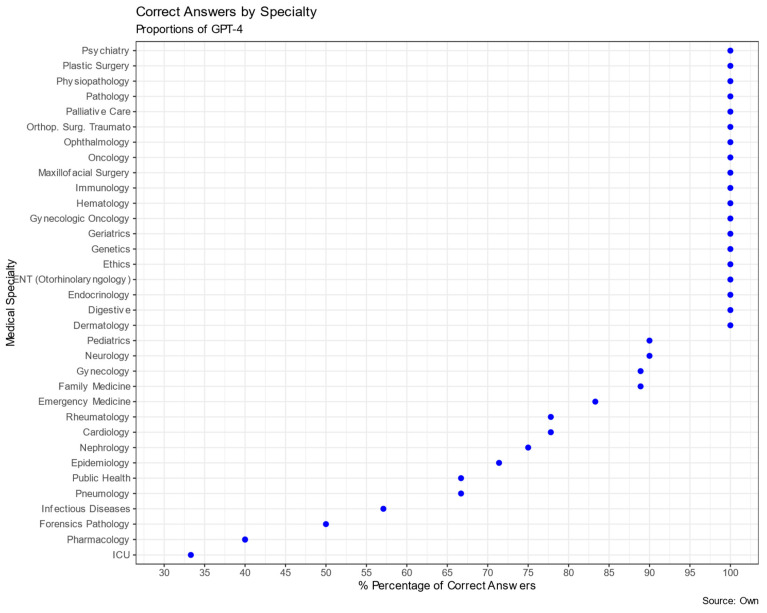
Proportions of GPT-4 correct answers by specialty.

The analysis of GPT-4’s performance on the MIR examination for Spanish physician residency program entrance across different medical specialties is presented in Table 3 and Figure 2. The total number of questions evaluated across all specialties was 182. GPT-4 exhibited varied success rates among the specialties. Specialties such as Pathology, Orthopedic Surgery and Traumatology, Dermatology, Digestive, Endocrinology, Plastic Surgery, Ethics, Physiopathology, Genetics, Geriatrics, Hematology, Immunology, Maxillofacial Surgery, Ophthalmology, Gynecologic Oncology, Oncology, ENT (Otorhinolaryngology), and Psychiatry yielded a 100% correct response rate. However, there were areas where GPT-4’s performance was significantly below expectations based on the adjusted residual analysis, such as in Pharmacology (40%, *p* < 0.001), Critical Care (33.3%, *p* < 0.01), and Infectious Diseases (57.1%, *p* < 0.05).

We observed consistency in evaluating GPT-4’s performance on the Spanish MIR examination across two submissions (Table 4). The first attempt resulted in an 86.8% accuracy rate, whereas the second attempt showed a slight increase, achieving an accuracy of 89%. The agreement between the two submissions, as measured by Cohen’s Kappa interrater, was substantial, with a value of 0.7418 (95% CI: 0.5973–0.8863), suggesting consistent performance across both trials. Moreover, the McNemar’s test revealed no statistically significant differences between the two attempts (*p* = 0.344).

Table 5 highlights GPT4’s responses to the MIR examination questions across varied sequence scenarios. In the original order, differences between the two attempts were notable in the number of wrongly presented values of 24 and 20. The first and second attempts demonstrated exact significances of 0.017 and 0.032, respectively, indicating statistically significant nonrandom patterns. When evaluating the randomized questions using the original sequence, a significance of 0.040 was observed, suggesting a similar nonrandom trend. However, a clear shift occurred when the randomized responses were assessed in their inherent order: the significance reached 1, pointing to a more randomized response pattern in this context. Considering the results, it is evident that GPT-4’s responses to the MIR examination’s questions manifest nonrandom patterns in both original sequence attempts and when the randomized sequence was evaluated against the original order. However, the model exhibits a trend towards randomness when the shuffled questions were assessed in their original order, highlighting the crucial role of question sequencing in dictating AI response tendencies.

We performed univariate and multivariate logistic regressions to determine if there were associations between the length of the questions and the success in answering the questions. The results are presented in Table 6. The number of words, characters, and tokens did not influence the examination performance of GPT-4.

We computed a Polynomial Logistic. Our results indicate that there is no statistically significant association between the length of the MIR examination questions (measured in hundreds of words, characters, or tokens) and the success rate of GPT-4 in answering them, both in terms of linear and quadratic effects (Table 7), indicating that the question length in the measured units does not significantly influence GPT-4’s performance on the MIR test.

Our analysis of the 182 questions from the Spanish MIR examination using GPT-4 resulted in an error rate of 13.2%, with 24 questions answered incorrectly. Delving into the potential implications of these errors, as described in Table 8 using the NCC MERP classification system, the “No error” rate was 5.5%. In contrast, the “Error no harm” category and the “Error harm” category, suggesting direct harm to patients if such errors occurred in real-world medical scenarios, resulted in a rate of 3.3%. The complete list of questions failed by GPT-4, with its answer and the correct answer, is found in Table S3.

Table 9 provides more granulated insights into the nature of these errors using the NCC MERP subcategories. The predominant error type was “A. capacity to cause an error”, which occurred in 41.7% of the errors with a rate of 5.5%. It is crucial to highlight the absence of errors in the gravest categories, “H. error required intervention to sustain life” and “I. error contributed to or resulted in death”, showing a 0% rate. This analysis underscores the importance of understanding the number of errors and their qualitative severity when assessing potential patient risks when failing to answer the MIR questions correctly.

We found an association between the potential risk of errors and specialty (*p* = 0.01). All the errors in Cardiology and Critical Care domain produced harm (*p* < 0.01), whereas the errors in Pneumology did not (*p* < 0.05) (Table 10). In further examining the variations in performance across different medical specialties, our analysis unveiled a nuanced landscape of GPT-4’s capabilities. The 13.2% error rate, representing 24 incorrectly answered questions, varied significantly across specialties, shedding light on the model’s strengths and areas for improvement. For instance, specialties such as cardiology and Critical Care saw a 100% rate of errors resulting in harm, while Pulmonary medicine saw no harmful errors. This highlights a discrepancy in the model’s ability to handle questions from different medical fields, indicating a need for targeted improvements. Additionally, it is paramount to note the absence of errors in the most severe categories, “H. Error required intervention to sustain life” and “I. Error contributed to or resulted in death”, reinforcing the model’s reliability in critical scenarios.

### Image Processing Capabilities

GPT-4 did not perform well when evaluating medical images. The success rate was twice as high when the questions were asked in English as in Spanish, 26.1% versus 13.0%, although these differences were not statistically significant (*p* = 0.250). When asked in English, the number of correct questions was the same as what would be expected by chance: six (Table 11). There was no association between the type of image and the correctness of the response in both English (*p* = 0.393) and Spanish (*p* = 0.330).

Table 12 presents the results, with or without using the images. There were no significant differences in the success rate of these questions independent of whether the image was uploaded in GPT-4.

## 4. Discussion

We analyzed the comparative performance between GPT-3.5 and GPT-4 in handling the Spanish MIR examination and provided essential insights into the advancements of LLM in medical knowledge comprehension and accuracy. The improved consistency demonstrated by GPT-4 across multiple attempts presents the refinements in training and underlying model architecture in the GPT-4 version. This superiority of GPT-4 over GPT-3.5 has also been shown in drug information queries [55,56].

Performance seems inversely related to the difficulty of questions, as shown in the Japanese National examination, where GPT-4 was 77.7% in easy questions and 73.3% in challenging questions [45] The length of the question had no significant bearing on GPT-4’s performance, which challenges our hypothesis that lengthier inputs might pose comprehension issues for the model.

The rate of success of GPT-4 (86.8%) was very close to that of the physician who scored the highest on the MIR examination (91.5%) [57]. The number of correct answers by GPT-4 could be higher because ten more questions were challenged, although the Ministry of Health did not accept them. The correct answer [58] to one of the contested questions coincided with the one chosen by GPT-4. In addition, GPT-4 failed in two questions that the academies considered unchallengeable.

A previous Spanish study on the same MIR examination of GPT3.5 obtained a lower score (54.8%) than ours [59]. One explanation of the difference could be the prompt used. In our study, we situated the questions in a context, whereas the other study asked the model to fill in the questions. How the prompt is formulated may affect the results.

Though relatively low, the GPT-4’s error rate of 13.2% should be a concern, especially when contextualized within the medical field where stakes are incredibly high. The categorization of these errors using the NCC MERP classification system reveals that while most of the errors might be benign, the existence of even a small percentage that could lead to patient harm emphasizes the importance of human oversight. Such oversight is an ethical obligation and could serve as a mitigation strategy to prevent the pitfalls of over-relying on AI recommendations without expert verification.

The presence of an error rate in GPT-4’s responses, particularly in high-stakes medical scenarios, necessitates carefully evaluating its applications and potential risks. The variability in performance across medical specialties points towards a nuanced understanding of the model’s capabilities, emphasizing the need for continued research and development to enhance its proficiency across all fields. The absence of errors in the gravest categories offers a reassuring perspective on the model’s reliability in critical situations. Nevertheless, the existence of errors that could lead to patient harm, however small the percentage, reinforces the imperative for stringent human oversight and the validation of AI-generated recommendations in healthcare. This approach ensures a balanced integration of AI in medical practice, optimizing its benefits while safeguarding patient safety and upholding the highest standards of medical care.

While GPT-4 and similar models represent significant advancements in AI-driven knowledge databases and comprehension, their function should remain supportive, especially in sensitive sectors like medicine. Medical practitioners must continue to utilize their training, experience, and intuition, using AI tools as complementary resources. In a study evaluating GPT-4’s clinical decision support using standardized clinical vignettes, the model achieved a 71.7% overall accuracy, excelling in final diagnosis tasks (76.9%) but showing lower performance in the initial differential diagnosis (60.3%) [60]. Another study on triage with clinical vignettes in ophthalmology found almost the same success rate as ophthalmologists in training: 93% of the model versus 95% of the residents. The same happened in predicting the correct diagnosis of common urinary diseases. ChatGPT had a higher accuracy rate (91%) in predicting the proper diagnosis of common urinary diseases than junior urology residents [61]. ChatGPT also has shown the ability to evaluate neuro-exams using established assessment scales [62]. It has outperformed medical students and neurosurgery residents on neurosurgery test examinations with a 79% success [63].

Another potential use of GPT-4 could be to answer patient questions. ChatGPT has been tested with real questions that patients ask physicians. The average accuracy scores (1 to 5) for questions about treatments, symptoms, and diagnostic tests were 3.9, 3.4, and 3.7, respectively [39]. GPT could be used in health education to help patients understand their diseases, treatment, and potential complications [64] and adapt their lifestyle to conditions such as metabolic syndrome or obesity [65,66].

Another use of GPT-4 could be to help physicians generate reports and make them comprehensible for patients [67] or even write them in another language. This has been shown especially in the field of Radiology [68,69]. The sequencing of the MIR examination questions is crucial in understanding GPT-4’s performance. Table 3 demonstrates that in evaluating GPT-4’s responses, it becomes clear that questions, when grouped by specialty, can create distinct patterns in the model’s response. For instance, GPT-4 managed notable success rates in Neurology (90.0%) and Cardiology (77.8%), whereas it grappled with specialties such as Pharmacology, achieving only a 40% success rate.

This specialty-based pattern becomes even more significant when the examination’s questions are shuffled, as observed in Table 4. When the questions were randomized, GPT-4’s performance, as measured by the Runs Test, changed. The model showed a nonrandom response pattern during the first three tests, with exact significance levels of 0.017, 0.042, and 0.040, suggesting that the model had difficulty with specific clusters of questions. However, when the randomized question order was evaluated as per the random sequence, the significance value rose sharply to 1.000, indicating a shift to a more random response pattern. This data implies that what we initially thought might be ‘model fatigue’ is more about the organization and sequence of the examination’s design. If the model found a series of questions challenging, it is likely because those questions were from a particularly tough specialty, which is not because of the sequence. This pattern can be misleading and interpreted as fatigue, mainly when questions of a challenging specialty are clustered together. Our analysis underlines the need to consider the nature of the questions and their internal organization and sequencing when evaluating AI performance, particularly with high-stakes exams. While intriguing, the notion of ‘model fatigue’ does not hold water when faced with the intricacies of the examination structure and the model’s response patterns. The term “model fatigue”, originally postulated in this research, pertains to the hypothesis that the performance of a machine learning model might degrade or show variation based on the sequence in which inputs are presented. This phenomenon is widely recognized in human cognition and performance, and investigating its presence in artificial intelligence models offers an intriguing avenue for exploration. Our study took a step forward in demystifying this concept, particularly in the context of large language models (LLM) answering medical examination questions.

The data collected and analyzed in our study shed light on the intricate relationship between question sequencing and model output. It was hypothesized that a pattern akin to fatigue might emerge, particularly when questions from challenging specialties were grouped, potentially overburdening the model and resulting in degraded performance. However, our results indicate that what might be perceived as ‘model fatigue’ is instead closely tied to the internal organization and sequence of the examination’s design.

It is crucial to distinguish between human fatigue, a physiological state, and the concept of ‘model fatigue,’ which we suggest might be better termed ‘model sensitivity to the input sequence.’ This distinction is essential because, unlike humans, LLMs do not experience tiredness or loss of focus over time. However, they can exhibit variations in performance based on the sequence of input, which might be misinterpreted as fatigue.

### 4.1. LLM Variable Responses

We found variability in GPT-4’s responses. Even when identical prompts are used, there can be some variability. This variability is an intriguing aspect of LLM design and offers a glimpse into the complexities of neural networks. Unlike deterministic systems (like traditional statistical packages), which consistently produce unchanging outputs for a given input, LLMs like GPT-4 operate in a probabilistic domain. The model identifies plausible outcomes for any given information and selects the most probable one. However, what is deemed “most probable” can vary significantly with each invocation due to the model’s stateless nature and inherent randomness.

Additionally, external factors like degradation in performance, technical glitches, or even subtle model updates can further introduce variability. The prompt’s ambiguity can also lead the model to be interpreted differently in separate instances. Though these variations might be seen as inconsistencies, they are a feature of the model’s dynamic and probabilistic architecture, setting it apart from traditional deterministic systems.

In the design phase, we had to decide between submitting the questions individually and raising the questions in blocks of 10. We chose to submit in 10-question blocks. This approach is faster and more efficient, especially when dealing with many questions. In addition, GPT-4 in August 2023 had a limitation of a maximum of 50 questions every 3 h. This posed several inconveniences: GPT-4 has a token limit (the exact number can depend on the specific version/configuration of the model). The model does not “see” the entire block if a block exceeds this limit. There could be potential carry-over effects: the context from one question could unintentionally influence the model’s response to a subsequent question, which might skew the results if not desired. Submitting the questions one by one could have the advantage of treating each question in isolation, eliminating potential contextual influence from previous questions, and allowing a more precise assessment of GPT-4’s performance on individual questions. It also ensures that each question is processed with the same amount of attention without being influenced by the “length” or complexity of prior questions in a session.

On the other hand, GPT-4 does not carry over any information from the previous question, which can be desirable if we want to ensure no “memory” effects between questions. Nevertheless, submitting one question isolated every time is more time-intensive than offering them in batches. Future research could test both strategies by submitting in batches or one by one and determine the best number of questions in the batches.

The results of the new feature using images were poor. Furthermore, the results were like those obtained when the same questions were prompted without using the images. This agrees with the literature suggesting that GPT-4 can solve issues involving images based on text without having access to the images [53]. At its current level of development, GPT-4 should not be used to evaluate medical images. Hopefully, future releases will improve training and image analysis capabilities.

### 4.2. Analysis of Errors

Although specialties such as Surgery and Pediatrics saw commendable success rates, GPT had a lower performance in Pharmacology, Critical Care, and Infectious Diseases, highlighting that there might be areas of medical knowledge that require a more nuanced understanding or perhaps more excellent representation in training data. Although many publications have studied the performance of GPT-4 in medical exams, few have broken down the responses according to medical specialties. In a study with Taiwan’s national exam taken in Chinese, GPT-4 performed well in Thoracic Medicine, whereas in our study, the performance was not good, with a success rate of 67% [44]. In the Taiwanese study, GPT-4 performed well in Gastroenterology and General Medicine. Something similar happened in our study, where the performance in Gastroenterology and Family Medicine was also high, with 100% and 89.9%, respectively. In the Taiwanese study, the performance in Infectious Diseases was low at 57.1%, which is the same rate as ours, 57.1%.

Wang’s study showed that ChatGPT performed well at answering questions on basic medicine, fundamental knowledge. However, it performed poorly on clinical questions designed to assess clinical thinking and reasoning skills, such as case analysis and treatment option selection, which is consistent with our findings of lower performance in Pharmacology-related questions [43]. In the same way, a study in which GPT-4 was presented with ten antidepressant-prescribing vignettes found that the model seems to recognize and utilize several standard strategies often used in psychopharmacology. However, due to some recommendations that may not be ideal, relying on LLM for psychopharmacologic treatment guidance without additional oversight remains risky [70].

There is no information available on how training with GPT-4 has been carried out, so it is not possible to know if the differences in performance in certain medical specialties are due to the documentation with which the model was trained, the characteristics of the questions, or due to the difficulties of the model with various specialties. However, in a scientific investigation involving GPT-4, researchers applied National Board of Medical Examiners (NBME) questions to the model and analyzed their explanations for the answers [71]. The findings indicated that correct responses frequently included information extending beyond the immediate scope of the question, with a noteworthy frequency (surpassing a threshold of *p* < 0.001). Such results imply a potential relationship between the model’s accuracy in answering questions and its ability to integrate the question with its extensive knowledge base. Pharmacology, infectious diseases, and critical care were the areas with many errors.

#### 4.2.1. Pharmacology Errors

The incorrect responses to the pharmacology questions can be linked to the challenges of AI in contextual medical understanding. For the management of bipolar disorder in a pregnant woman, GPT-4 incorrectly recommended lithium instead of olanzapine. This error may arise from an over-reliance on historical data where lithium was a standard recommendation, not accounting for more recent research that favors olanzapine due to its better safety profile in pregnancy [72,73,74,75,76,77,78,79]. Furthermore, an old review from 2006 found that olanzapine was associated with a higher risk of metabolic complications in pregnant women, but the data on the safety of these compounds during breastfeeding in this study are also noted to be anecdotal [79].

Regarding the recommendation for NSAID use in a patient with duodenal ulcer and high cardiovascular risk, GPT-4 incorrectly favored celecoxib over naproxen. This choice may reflect a misjudgment in balancing gastrointestinal safety with cardiovascular risk; newer evidence suggests that naproxen may have a more favorable cardiovascular risk profile [80,81,82,83,84].

Lastly, in advising a diabetes treatment for a patient with a new episode of heart failure, GPT-4’s suggestion to use canagliflozin over pioglitazone overlooks the fact that pioglitazone, known for its fluid retention side effect, is contraindicated in heart failure patients—a significant error reflecting a possible gap in the model’s integration of drug contraindications with comorbid conditions [85,86,87].

The consequences of these pharmacological inaccuracies vary in severity. The recommendation of lithium for treating bipolar disorder during pregnancy (for the question about bipolar disorder treatment safety in pregnancy) could lead to Category D error consequences, where monitoring and potential intervention would be necessary to prevent fetal harm due to lithium’s known teratogenic risks. The second error, which involved the selection of an NSAID for a patient with cardiovascular risk (the question about NSAID selection for a patient with duodenal ulcer and ischemic heart disease), falls into Category C, as the decision could lead to a medication reaching the patient without causing harm—celecoxib does have some cardiovascular risk, but not significantly higher than other NSAIDs, and in some cases might be considered a safer alternative for gastrointestinal issues. The third mistake, recommending canagliflozin in a patient with heart failure (in the question about optimizing diabetes treatment post-heart failure episode), may lead to Category E consequences where the patient could experience temporary harm due to the drug’s potential to worsen heart failure, requiring immediate intervention. These scenarios illustrate that AI can provide valuable insights but cannot replace the nuanced, patient-specific judgments of healthcare professionals guided by the most current clinical evidence.

#### 4.2.2. Infectious Diseases Errors

In the Infectious disease questions, the errors in the responses provided by GPT-4 can be attributed to the complexity of clinical scenarios and the nuances of medical guidelines, which may not be fully encapsulated in the training data. For instance, the recommendation of post-exposure prophylaxis for HIV for 28 days despite the patient’s contact with an HIV-positive person with an undetectable viral load may reflect an outdated understanding of transmission risks. Updated guidelines recognize the minimal risk in such cases, hence the incorrect suggestion of prophylaxis [88,89,90,91]. The unnecessary post-exposure prophylaxis is a Category C error, where the patient is exposed to unnecessary medication with possible side effects but without direct harm.

In another question, the error in identifying the clinical picture as diagnostic overlooks the need for confirmatory laboratory tests despite characteristic symptoms of group A Streptococcus pyogenes infection, which can mimic other conditions. The reliance on clinical presentation without confirmatory testing could lead to a Category D error, where inappropriate treatment might be given, requiring further monitoring to ensure that no harm occurs.

A complex clinical situation where a 72-year-old patient with diabetes and hypertension, who had a history of fever and malaise accompanied by a new cardiac murmur and positive blood cultures for methicillin-resistant Staphylococcus aureus, was inadequately advised by GPT-4 to add IV ceftaroline and diuretics for treatment, rather than the necessary urgent valve replacement surgery indicated by continued fever and signs of heart failure despite ongoing antibiotic therapy. The failure to prioritize valve replacement surgery in the face of persistent fever and heart failure symptoms after appropriate antibiotic therapy could result in a Category E error, where a delay in the correct surgical intervention may contribute to temporary harm, requiring additional treatment to address the complications of incorrect initial management.

#### 4.2.3. Critical Care Errors

Regarding the erroneous responses to critical care questions, the mistakes can be attributed to the complexity of medical decision making and the inherent limitations of an AI in interpreting clinical context. In a question about the positional technique in a patient with legionella with acute respiratory distress syndrome, the incorrect response suggested by the AI was that the prone position during invasive mechanical ventilation requires deep sedation [92,93]. This reflects a misunderstanding of critical care protocols, where deep sedation is not always a requirement and is determined on a case-by-case basis. The consequences of such errors are significant if they occur in a real-life setting. In this case, an error is classed as Category F because it could have led to unnecessary deep sedation, potentially resulting in prolonged hospitalization due to complications from over-sedation, such as hemodynamic instability or prolonged weaning from mechanical ventilation. This error type underscores the importance of accurate, context-driven clinical decision-making, particularly in critical care settings.

In a question concerning the diagnostic orientation and therapeutic attitude following a tracheostomy in a COVID-19 patient, the AI incorrectly identified Guillain–Barré Syndrome as the initial diagnosis and recommended confirmatory tests. This error likely stems from the AI’s interpretation of motor deficits and hyporeflexia as indicative of Guillain–Barré Syndrome without sufficiently considering the context of recent critical illness, which is more suggestive of critical illness myopathy. The error was classified as a Category D because it could have caused an incorrect diagnostic path to be pursued, necessitating additional monitoring and interventions to ensure no harm came to the patient. Misdiagnosing critical illness myopathy such as Guillain–Barré Syndrome could lead to unnecessary tests and delay the appropriate management, which includes intensive respiratory and motor rehabilitation [94,95,96,97].

#### 4.2.4. Cardiovascular Errors

There were two cardiovascular errors. The first one was a case of a 75-year-old male with symptoms indicative of aortic stenosis. GPT-4 provided an incorrect answer, suggesting surgical replacement of the aortic valve rather than the correct percutaneous aortic valve implantation (TAVI). The error stems from a misinterpretation of the clinical context provided, where a patient with intermediate surgical risk and elevated creatinine levels would be better suited for a less invasive procedure like TAVI [98,99]. This misjudgment could have been due to an oversight in assessing all patient risk factors, including age, symptoms, and renal function, which are pivotal in determining the most appropriate therapeutic approach. The consequences of these errors, if they had occurred in a clinical setting, could be significant because selecting a surgical aortic valve replacement over TAVI in a patient with several risk factors for surgery could result in permanent harm (Category G), given the higher morbidity and mortality associated with open-heart surgery compared to the less invasive TAVI, particularly in a patient with reduced renal function [100].

The second error involved a patient with swelling and heaviness in her right arm, lasting 72 h, and edema of the right upper limb with dilated veins in the pectoral region. GPT-4 incorrectly affirmed that phlebography is necessary to confirm axillary–subclavian venous thrombosis. This recommendation is inconsistent with current clinical guidelines prioritizing non-invasive methods for initial diagnosis, given the availability and effectiveness of Color Doppler ultrasound and angio-CT [101,102]. The error seems to stem from an overreliance on less current diagnostic approaches that were once standard but are now typically reserved for cases where initial non-invasive tests are inconclusive or surgical intervention is being considered [103]. The consequences of such a diagnostic error in a real-world scenario could be significant, falling under the NCC MERP classification of Category E, wherein the error could temporarily harm the patient requiring intervention. If a patient undergoes an unnecessary phlebography based on this incorrect advice, it could lead to potential complications such as allergic reactions to contrast dye, phlebitis, or even more severe complications like venous thromboembolism [104,105]. Moreover, the invasive nature of phlebography compared to non-invasive options would also pose additional psychological stress for the patient. Beyond the direct health risks, unnecessary healthcare costs and resource utilization could impact the patient and the healthcare system.

#### 4.2.5. Errors in Obstetrics and Gynecology

GPT-4 provided an incorrect answer to the indication for applying vacuum extraction during delivery. The correct scenario for applying a vacuum to shorten the expulsive period is when fetal bradycardia occurs that does not recover after a contraction ends, and the posterior fontanel is past the third Hodge plane [106,107]. This suggests that the baby is in the birth canal but is experiencing distress, necessitating prompt delivery [108]. GPT-4’s incorrect response was to apply the vacuum after 2 h of full dilation when the baby’s position remains at the second Hodge plane with maternal pushing. The error likely originated from an over-reliance on a fixed time criterion without adequately weighing the critical factor of fetal distress, which is a more immediate and decisive indication for intervention. Another potential source of errors could be that GPT-4 based its decision on the guidance from the National Institute for Health and Care Excellence (NICE), in which vacuum extraction is recommended after 2 h without epidural and 3 h with epidural during labor [109], noting that in Spain, most women receive epidural anesthesia, and that NICE uses a different nomenclature for referring to the fetal head station compared to the terminology commonly used in Spain [110]. The potential consequences of this error could have clinical implications. If the incorrect criterion were applied, the use of vacuum extraction might be unnecessarily delayed while waiting for the specified 2 h period of full dilation, despite the presence of fetal distress. This delay could lead to a scenario where there is a risk of harm to the fetus that requires monitoring or intervention to preclude harm (Category D).

### 4.3. Limitations of the Study

Our study has several limitations. First, the number of questions in each specialty is limited, so the confidence intervals for GPT success are enormous. Furthermore, in the future, the evaluation of more tests to estimate the success rate more accurately is needed. It would also be interesting to ask the model for the reasons for choosing the wrong answer to determine the causes of the failures. Progressively, it could be tested as a diagnostic aid in those specialties with higher success rates.

Second, our study results demonstrate that LLM models can show highly variable accuracy across different domains. Our study corroborates earlier observations on the performance of GPT-4 in the MIR examination across different medical specialties and shows promise when applied in various medical disciplines such as allergology, dermatology, and radiology [1,2,3]. GPT-4 achieved a perfect score in specialties including Dermatology, aligning with prior literature highlighting its efficacy in that field. Amazingly, in our study, GPT-4 chose the correct answer in 100% of the Gastroenterology (digestive) questions, but another study failed the Gastroenterology examination, not reaching the score of 70% necessary to pass the test [38]. The variability in performance across specialties does support our initial hypothesis, indicating that while GPT-4 shows exemplary results in some areas, it has potential weaknesses in others, such as Pharmacology and Critical Care. This differential performance suggests that navigating specialized medical examinations like the MIR demands a broader and more nuanced knowledge base. Understanding where AI tools like GPT-4 excel and how they can best complement human expertise is crucial.

Third, the obsolescence index applies to LLM searches and measures how quickly information becomes outdated. For instance, the obsolescence index for medical textbooks of Internal Medicine is around five years, meaning that a fresh out-of-print book has five years of relevant information. On the other hand, with the time considered for writing, peer review, and journal submission, scientific papers may have an obsolescence index of one year [111,112]. This rapid turnover in medical knowledge underscores the importance of regular updates to AI models to ensure their recommendations remain current and clinically relevant. A testament to the vast and ever-growing body of medical information is a recent search by the authors on PubMed on 19 August 2023, which yielded a staggering 387,974 papers related to COVID-19 alone. With search terms like “coronavirus”, “COVID”, “COVID-19”, and “SARS-CoV-2”, this immense volume of information is impossible for any individual physician to sift through comprehensively. However, this is precisely where artificial intelligence systems can excel. By rapidly processing and analyzing vast datasets, AI can help synthesize pertinent information and insights, ensuring that medical professionals can be assisted with the most up-to-date knowledge. At the same time, AI can provide quick answers. However, a judicious approach is essential.

Despite its advanced features, GPT-4 shares certain constraints with its predecessors in the GPT series. Specifically, it is not entirely dependable, as it can generate inaccurate or fictional content, referred to as “hallucinations”. A hallucination is a factual inaccuracy that appears to be scientifically plausible [113,114,115] but is a fabrication [116]. Hallucinations also happen when GPT-4 produces nonsensical reasoning [117]. GPT can also generate fake bibliographic references [118,119]. For example, one study about kidney transplantation found that ChatGPT failed to provide references for any of the scientific data it provided regarding kidney transplants, and when requested for references, it provided inaccurate ones [120]. Due to that, it is always essential to verify the information provided by ChatGPT [121].

Additionally, its ability to comprehend and process text is confined to a restricted context window, and it cannot acquire knowledge from past interactions [50,122,123,124]. Physicians must remain vigilant, cross-referencing recommendations, especially in areas where the AI’s performance has shown high variability, and be mindful of potential “hallucinations” or inaccuracies in AI outputs. In addition, there is some variability in the answers when they are sent several times to the model, so it is recommended in research to independently send the questions several times to the model and consider the range of answers [41].

Fourth, LLMs have yet been shown to evaluate or improve hospital-specific activities such as medical documentation, e.g., coding causes of hospitalization or death using the International Classification of Diseases, infection control, and reviewing medical records to detect whether a patient has had a healthcare-associated infection. In the continually evolving landscape of Medicine, the potential applications of advanced tools like GPT in clinical practice are manifold. ChatGPT is adept at handling real-world patient queries [4]. Patients can also use ChatGPT to seek information when they fear sharing their doubts on sensible issues with their physicians [125]. ChatGPT, first unveiled in September 2021, offers a myriad of uses, from aiding physicians in differential diagnosis and treatment recommendations to facilitating patient education and streamlining telemedicine consultations. It can also assist medical research, providing rapid summaries of the latest studies or advancements in specific domains.

Fifth, the study focused solely on one of the Spanish MIR medical examinations. In the future, it would be interesting to repeat the study for more years and with other health professions like nursing, pharmacy, and psychology.

### 4.4. Future Developments of IA in Medicine

In reflecting upon the implications of the recent healthcare advancements in artificial intelligence (AI), our discussion draws upon the role of medical staff engagement in developing and accepting AI tools. A study that explored the increasing integration of artificial intelligence (AI) in healthcare focusing on AI-assisted diagnostic and decision-making tools (AI-IDT and AI-ADT) found that medical staff’s active participation in AI development significantly boosts their acceptance of these tools, influenced by cognitive improvements in AI self-efficacy and reductions in AI anxiety [126]. While AI demonstrates the potential to ease staff workload and enhance healthcare processes, persistent challenges in motivating staff acceptance need to be addressed [127].

The integration of AI into clinical medical imaging is fraught with challenges despite deep neural networks excelling at analyzing high-stakes images [128]. Common challenges include substantial computational needs, tedious data annotation, domain shifting, and a lack of explainability. Data collection is especially problematic in the current era of stringent data privacy laws. While researchers are focused on developing larger, more accurate networks, physicians and legal frameworks are more concerned with ensuring that these networks are trustworthy, ethical, and transparent in their decision-making processes [129]. In medical imaging, AI may enhance safety and reduce costs by aiding patient navigation and minimizing radiation exposure risks to patients and nurses in the Nuclear Medicine Department [130]. The medical imaging capabilities of GPT-4 are currently minimal and should not be used in its current state. It should be kept in mind that the technology is still in its infancy. The image handling and interpretation capabilities and their integration with GPT-4 or other LLMs may be improved in successive versions.

### 4.5. GPT-4 in Teaching and Examinations

While it might be conjectured that GPT-4 could be utilized for cheating on medical examinations, including the MIR, and at medical schools, this is not true for several reasons. First and foremost, the examination protocols for the MIR explicitly prohibit the use of mobile phones or any other devices capable of storing information or facilitating communication, either through voice or data, within a strict time frame of four hours and thirty minutes [49]. These regulations are rigorously enforced to maintain the integrity of the exam. Universities and institutions have strict policies and penalties for students caught cheating [131,132]. Some universities impose sanctions at the educator’s discretion, often resulting in automatic failure, while other schools suspend students caught cheating outright.

Furthermore, many universities ask their student at the registration to sign a declaration of academic honesty, pledging not to cheat [133]. It is paramount to acknowledge that while cheating on an exam may not be a criminal offense, it is nevertheless an unethical act with severe academic repercussions. Thus, the structured measures are robust enough to significantly mitigate the risk of using tools like GPT-4 to gain an unfair advantage in medical exams.

Moreover, the landscape of examinations is evolving, with many being conducted in computer labs using university-provided computers on platforms such as Moodle. These digital environments are designed with security in mind; they typically operate in a locked-down mode that blocks all internet access during the examination period. Additionally, surveillance mechanisms allow proctors to monitor if a student attempts to switch screens or access unauthorized material. Moodle, a widely adopted learning management system [134], has “Safe Exam Browser” capabilities that restrict browsing and lock down the system, ensuring that students can only access exam materials, reducing the potential for misconduct, including the illicit use of GPT-4 or other online resources, thus maintaining the integrity of the examination process. These stringent measures, both traditional and technological, work to prevent cheating and uphold the fairness and academic standards of medical examinations. Other universities use different types of proctoring software, and some universities consider using during examinations frequency inhibitors, formerly only used by the police and the army.

Two distinct approaches regarding using GPT-4 for completing assignments and exercises are being considered. One strategy involves prohibiting its use and software implementation to detect whether AI has generated content. The other approach, which we advocate, is to permit the use of GPT-4 but require students to include an appendix in their work detailing the prompts given and the outputs received from GPT-4. We advocate for the latter method as we believe that, soon, large language models (LLMs) like GPT-4 will be utilized in clinical settings as a tool for helping with diagnosis or treatments, and students must be made aware of their capabilities and limitations. They must also be cognizant of the ethical and legal issues involved, as ultimately, the clinician is responsible for the final decisions, not the software. Physicians must ensure that their choices based on AI’s suggestions are appropriate and medically sound. Integrating LLMs into medical education transparently prepares future doctors to integrate AI tools effectively and ethically into their practice, fostering an environment where the technology is seen as an aid rather than a replacement for clinician judgment.

### 4.6. Implications of This Study

The prospects of integrating AI tools like GPT into Medicine are promising, and a collaborative approach remains paramount, where AI can be used to complement rather than endeavor to replace human expertise. Future research should explore mechanisms to integrate AI feedback with human expertise, potentially through hybrid decision-making systems, to harness the strengths of both systems. ChatGPT could be used by physicians as a diagnostic assistance tool, helping physicians with differential diagnoses [32] and helping personalize treatments based on existing evidence and scientific literature [32]. As technology evolves and refined models emerge, continuous evaluation will be critical, as this study did. Such evaluations ensure that while we tap into the immense potential of AI, we remain grounded in the core objective: enhancing patient care and medical outcomes. Physicians are routinely faced with life-and-death decisions for their patients, choices that are weighted with profound moral and ethical considerations. Such critical decisions cannot be relegated to AI systems, which often function as “black boxes” with opaque inner workings and unclear training methodologies [135]. The exact nature of the data and documentation used in their training processes is not always fully transparent, which can raise questions about the reliability and trustworthiness of their outputs [26], which could delay AI deployment in clinical settings [136]. AI can reduce physicians’ risks but poses serious risks [27]. Therefore, these pivotal decisions must be made by humans, who can interpret the AI’s recommendations through the lens of bioethical principles. While AI systems can serve as valuable tools in assisting with diagnosis, formulating differential diagnoses, and suggesting treatment options, they remain adjuncts to human expertise. Ultimately, a human being—a trained medical professional—must bear the responsibility for any clinical decision. By ensuring that AI does not overstep its supportive role, we safeguard the essential human element in healthcare, ensuring that empathy, ethical standards, and professional accountability govern patient care [3]. There are also issues related to data protection and privacy [135].

We need to recognize that the latest advances in medical and computer technology are raising many questions relative to the ethics of real-world care and pledge to support ethical medical technologies with the goal of *“Primum non nocere”* [137].

## 5. Conclusions

This study illuminates the advanced performance of LLMs and the differences between GPT-3.5 and GPT-4 in addressing the Spanish MIR examination for medical specialization. Significantly, GPT-4 displayed a superior accuracy rate in Spanish and English, consistent across multiple submissions. Despite the impressive success rates across various specialties, some areas like Pharmacology, Critical Care, and Infectious Diseases revealed notable shortcomings. Interestingly, when quantified in terms of words, characters, or tokens, the question length did not influence GPT-4’s performance. While the model’s error rate stood at 13.2%, the potential implications of these mistakes, when framed using the NCC MERP classification system, point towards a low percentage of errors that could result in direct harm. The medical imaging capabilities of GPT are currently very limited and should not be used. AI models like GPT-4 exhibit strong capabilities, but their application in medicine requires meticulous scrutiny and emphasizes further improvements in LLMs before they can be reliably deployed in medical settings for decision-making support.

## Figures and Tables

**Table 1:** Comparison of correct response proportions for input in Spanish and English: GPT-3.5 vs. GPT-4, with McNemar test results.

Language	GPT-4 % (95% CI)	GPT-3.5 % (95% CI)	Sig **
Spanish	86.81 (81.13–90.98)	63.18 (55.98 -69.85)	<0.001
English	87.91 (82.38–91.88)	66.48 (59.35 –72.94)	<0.001
Sig *	0.824	0.441	N = 182

N = 182 * comparison between languages within versions; ** comparison between versions within languages.

**Table 2:** Comparison of correct response proportions for MIR examination theoretical and practical questions.

	GPT-4 % Correct Answers (N = 182)	Significance †
Type of questions		0.927
Theoretical	87.1% (85)	
Practical	86.6% (97)	

N = Total number of questions; † Chi square.

**Table 3:** Comparison of correct response proportions of GPT-4 for MIR examination in Spanish by specialty.

Specialty	% Correct	N	Specialty	% Correct	N
Pathology	100%	1	Forensics & Legal Medicine	50.0%	2
Cardiology	77.8%	9	Family Medicine	88.9%	9
Orthopedic Surgery and Traumatology	100%	10	Nephrology	75.0%	4
dermatology	100%	1	Pneumology	66.7%	6
digestive	100%	6	Neurology	90.0%	10
endocrinology	100%	8	Ophthalmology	100%	3
epidemiology	71.4%	7	gynecologic oncology	100%	2
Plastic Surgery	100%	2	Oncology	100%	2
Ethics	100%	4	ENT (Otorhinolaryngology)	100%	3
pharmacology	40% ***	5	palliative care	100%	2
physiopathology	100%	7	Pediatrics	90.0%	10
genetics	100%	2	Public Health	66.7%	3
geriatrics	100%	6	Psychiatry	100%	8
gynecology	88.9%	9	Rheumatology	77.8%	9
hematology	100%	5	Critical Care	33.3% **	3
infectious diseases	57.1% *	7	emergency medicine	83.3%	6
immunology	100%	7			
Maxillofacial surgery	100%	2	TOTAL		182

Adjusted residual analysis. * *p* < 0.05; ** *p* < 0.01; *** *p* < 0.001.

**Table 4:** Comparison of correct response proportions for first and second attempts in Spanish using GPT-4, with McNemar test results.

	Second Attempt		
First Attempt	Wrong	Correct	Total
Wrong	17	7	24
Correct	3	155	158
Total	20	162	182

McNemar test. *p*_(exact)_ = 0.344; Cohen’s Kappa = 0.74 (95% CI: 0.59–0.88).

**Table 5:** Comparison of GPT-4’s responses to MIR examination questions: effects of euestion order on Runs Test outcomes.

	Original Question Sequence		Random Question Sequence	
Test Scenario	1st Attempt	2nd Attempt	Evaluated with the Original Sequence	Evaluated with the Random Order
Test Value Median	1	1	1	1
Wrong answers	24	20	23	23
Correct answers	158	162	159	159
Total Questions	182	182	182	182
Number of Runs	35	31	35	41
Z	−2.507	−2.148	−2.097	−0.063
Exact Significance (2-tailed)	0.017	0.032	0.040	1.000

**Table 6:** Univariate and multivariate logistic regression of GPT-4’s responses to MIR examination questions: effects of number of words, characters, and tokens (all in hundreds).

	Univariate Logistic Regression		Multivariate Logistic Regression	
Length of the Question	OR (95% CI)	*p*	OR (95% CI)	*p*
Number of Words *	1.82 (95% CI 0.51–6.47)	0.351	1.33 (95% CI 0.03–53.57)	0.880
Number of Characters *	1.09 (95% CI 0.90–1.34)	0.370	1.70 (95% CI 0.01–259.69)	0.835
Number of Tokens *	1.30 (95% CI 0.74–2.33)	0.361	0.92 (95% CI 0.24–3.45)	0.896

* In hundreds.

**Table 7:** Univariate Polynomial Logistic Regression of GPT-4’s responses to MIR examination questions: effects of number of words, square words, characters, square characters, tokens and square tokens (all in hundreds).

	Univariate Polynomial Logistic Regression	
Length of the Question	OR (95% CI)	*p*
Words		
words	1.86 (0.51–6.80)	0.344
Words ^2^	0.84 (0.06–11.10)	0.893
Characters		
Characters	1.11 (0.91–1.36)	0.293
Characters ^2^	0.983 (0.92–1.05)	0.588
Tokens		
Tokens	1.38 (0.77–2.50)	0.283
Tokens ^2^	0.88 (0.55–1.41)	0.597

**Table 8:** Distribution and potential risk of errors identified using the NCC MERP classification system categories.

Type of Error	N	%	Rate % (95% CI)
1. No error	10	41.7	5.5 (3.0–9.8)
2. Error no harm	8	33.3	4.4 (2.2–8.4)
3. Error harm	6	25.0	3.3 (1.5–7.0)
Total Incorrect Answers	24	100	
Correct Answers	158		
Total Questions	182		

**Table 9:** Distribution and potential risk of errors were identified using the NCC MERP classification system subcategories.

Type of Error	N	%	Rate % (95% CI)
- A. Capacity to cause error.	10	41.7	5.5 (3.0–9.8)
- B. Error did not reach the patient.	1	4.4	5.4 (0.9–3.0)
- C. Error reached patient did not cause harm.	3	12.5	1.6 (0.6–4.7)
- D. Error reached the patient and required monitoring	4	16.7	2.2 (0.9–5.5)
- E. Error temporary harm and required intervention.	2	8.3	1.1 (0.3–3.9)
- F. Error required hospitalization.	2	8.3	1.1 (0.3–3.9)
- G. Error resulted in permanent patient harm.	2	8.3	1.1 (0.3–3.9)
- H. Error required intervention to sustain life.	0	0	0 (0–2.0)
- I. Error contributed to or resulted in the death	0	0	0 (0–2.0)
Total Incorrect Answers	24	100	
Correct Answers	158		
Total Questions	182		

**Table 10:** Distribution of potential risk of errors identified using the NCC MERP classification by specialty and system categories.

Specialty	No Error N (%)	No Harm N (%)	Harm N (%)	Total
Cardiovascular	0	0	2 ** (100%)	2 (100%)
Epidemiology	2 (100%)	0	0	2 (100%)
Pharmacology	0	2 (66.6%)	1 (33.3%)	3 (100%)
Gynecology	0	1 (100%)	0	1 (100%)
Infectious Diseases	0	2 (66.6%)	1 (33.3%)	3 (100%)
Forensics & Legal Medicine	1 (100%)	0	0	1 (100%)
Family Medicine	1 (100%)	0	0	1 (100%)
Nephrology	1 (100%)	0	0	1 (100%)
Pneumology	0	2 * (100%)	0	2 (100%)
Neurology	1 (100%)	0	0	1 (100%)
Pediatrics	1 (100%)	0	0	1 (100%)
Public Health	0	1 (100%)	0	1 (100%)
Rheumatology	2 (100%)	0	0	2 (100%)
Critical Care	0	0	2 ** (100%)	2 (100%)
Emergency medicine	1 (100%)	0	0	1 (100%)
TOTAL	10 (41.7%)	8 (33.3%)	6 (25%)	24 (100%)

X^2^ = 38.667; df = 28; *p*_(exact)_ = 0.011. Adjusted standardized residuals, * *p* < 0.05 and ** *p* < 0.01.

**Table 11:** Comparison of correct response proportions for questions with images formulated in Spanish and English using McNemar test.

Language	Number of Questions	Number of Correct Answers	% (95% CI)
Spanish	23	3	13.0 (4.5–32.1)
English	23	6	26.1 (12.6–46.5)

*p* = 0.250.

**Table 12:** Comparison of correct response proportions for questions with Images formulated in Spanish and English with and without uploading images.

Language N= 24	% Correct Answers Using Images	% Correct Answers No Using Images	*p* *
Spanish	13.0%	17.4%	1.000
English	26.1%	21.7%	1.000
*p* *	0.250	0.625	-

* McNemar Test.

## Data Availability

The database can be obtained from the authors upon reasonable request.

## Supplementary Materials

The following supporting information can be downloaded at https://www.mdpi.com/article/10.3390/clinpract13060130/s1, Table S1: English Translation of the MIR Examination. Table S2: Answer to MIR examination questions. Table S3: Questions failed by GPT-4 in the MIR examination, indicating the correct answer, the wrong answer, and the Error classification. Table S4: Images and English translation of the questions with images.
