# Supplementary material for: Evaluating the Efficacy of ChatGPT in Navigating the Spanish Medical Residency Entrance Examination (MIR): Promising Horizons for AI in Clinical Medicine
Source: Clin Pract. 2023 Nov 20;13(6):1460–87. doi: 10.3390/clinpract13060130 (PMC10660543; doi:10.3390/clinpract13060130)
Supplement: Supplementary file 1 [file clinpract-13-00130-s001.zip › Table S1 English Translation of the MIR Examination.pdf]

Table S1. English Translation of the MIR Examination

1- In a patient with nephrotic syndrome, it is possible to find one of the following plasma alterations in the lipoprotein profile:

1. Reduction of very low-density lipoproteins (VLDL).

2. Increase in apolipoprotein B-100.

3. Increase in apolipoprotein A-I.

4. Increase in lipoprotein lipase (LPL) activity.

2- In relation to nitrogen compound metabolism, skeletal muscle is able to:

1. Synthesize urea to a limited extent in cases of liver dysfunction to counteract the toxicity of ammonia.

2. Use alpha-keto acids derived from branched amino acids as an energy source.

3. Produce alanine by transamination from pyruvate and aspartic acid, especially in certain fasting situations.

4. Produce creatine using glycine, ATP, and ammonia to obtain creatine-phosphate as an energy reserve and enable its use during exercise.

3- In type Ia glycogenosis (von Gierke's disease) due to glucose-6-phosphatase deficiency, it is characteristic:

1. Increased muscle glycogen.

2. Hypouricemia due to reduced renal reabsorption of uric acid.

3. Increased plasma lactate.

4. Decreased hepatic synthesis of triglycerides.

4- Regarding the pressures that cause the entry and exit of air in the lungs of a healthy person, indicate the INCORRECT statement:

1. The continuous aspiration of excess fluids into the lymphatic ducts maintains a slight negative pressure between the visceral surface of the lung and the parietal pleural surface of the thoracic cavity.

2. During normal inspiration, pleural pressure becomes more negative.

3. During normal expiration, alveolar pressure decreases compared to atmospheric pressure.

4. Transpulmonary pressure (alveolar pressure minus pleural pressure) tends to collapse the lungs during the complete respiratory cycle.

5- Turbulent blood flow tends to increase in direct proportion to all the following factors EXCEPT ONE. Indicate which:

1. Blood viscosity.

2. Blood flow speed.

3. Blood vessel diameter.

4. Blood density.

6- Regarding the regulation of fluid exchange and osmotic balance between intracellular and extracellular fluids in a physiological situation, indicate the INCORRECT statement:

1. The distribution of fluid between intracellular and extracellular compartments is mainly determined by the osmotic effect of the smallest ions.

2. About 80% of the total osmolarity of interstitial fluid and plasma is due to sodium and chlorine ions.

3. About 50% of osmolarity in intracellular fluid is due to calcium ions.

4. Relatively small changes in the concentration of non-diffusible solutes in extracellular fluid can cause large changes in cell volume.

7- Point out the correct answer regarding renal reabsorption of sodium:

1. Sodium is reabsorbed in the ascending loop of Henle without reabsorbing water.

2. 20% of the sodium is reabsorbed in the collecting tubule.

3. The highest sodium reabsorption occurs in the distal tubule.

4. Sodium reabsorption in the proximal tubule is mediated by aldosterone.

8- How is the progressively earlier onset and the increase in the severity of the clinical manifestations of a disease over successive generations called?

1. Variable penetrance.

2. Genetic anticipation.

3. Allelic anticipation.

4. Complete penetrance.

9- In relation to the role of immunoglobulin A (IgA) in defense against pathogens, indicate the INCORRECT answer:

1. IgA activates the classical pathway of the complement, contributing to the elimination of extracellular bacteria.

2. IgA is passed from the mother to the newborn during breastfeeding, contributing to the protection of the neonate's mucosa.

3. IgA can cross the epithelial barrier of mucous membranes by binding to the poly-Ig receptor (pIgR), being the most abundant immunoglobulin in mucosa.

4. Selective IgA deficiency is the most common immunodeficiency (approximately 1:400), and most cases are asymptomatic.

10- Among the new protective measures against COVID-19 is the use of therapies based on monoclonal antibodies against the S (spike) protein of SARS-CoV-2 (tixagevimab and cilgavimab). This treatment:

1. Is a form of active immunization whose outcome depends on the activation of memory lymphocytes.

2. Is indicated in immunocompromised patients, such as those with organ transplants.

3. Provides protection for an approximate period of two years.

4. Induces immunological memory based on the activation of B lymphocytes.

11- Regulatory T cells (CD4+, CD25+, FoxP3+) are frequently found in the infiltrate of solid tumors. These cells:

1. Are considered markers of a good prognosis in tumor development.

2. Contribute to antitumor immune defense.

3. Produce interferon gamma that contributes to the activation of cytotoxic lymphocytes.

4. Produce interleukin-10 that contributes to an immunosuppressive tumor microenvironment.

12- The hyper-IgM syndrome, a primary immunodeficiency associated with different mutations in CD40 or its ligand CD40L, is characterized because:

1. Patients have elevated levels of IgM while maintaining normal levels of IgG and IgA in serum.

2. There is a defect in the differentiation of B lymphocytes in the bone marrow with an increase in immature B lymphocytes that express the intracytoplasmic mu chain.

3. There is a defect in the collaboration process between T and B lymphocytes necessary for the immunoglobulins isotype switch.

4. There is a defect in the process of rearranging the V genes of immunoglobulins.

13- Among the advances in antitumor immunotherapy is the use of T cells modified to express a CAR receptor (chimeric antigen receptor), called CAR-T. Regarding CAR-T cells for the treatment of lymphomas and leukemias that express CD19, indicate the INCORRECT answer:

1. The recognition element of the CAR receptor is based on anti-CD19 antibody variable fragments (single-chain variable fragments, scFvs).

2. The CAR receptor has an intracellular domain responsible for signal transmission.

3. One of the serious side effects of this treatment is the cytokine release syndrome.

4. The antigen recognition by the CAR receptor depends on the class I histocompatibility molecules (HLA) in the tumor cell.

Here are the translations of the given questions and answers:

14- A 50-year-old beekeeper who experiences severe systemic reactions (anaphylaxis) after bee stings and who tests positive for Apis mellifera venom through skin tests in intradermal reaction and specific IgE in serum. What etiological treatment is indicated?

1. Training the patient in the use of adrenaline auto-injectors.

2. Educating the patient in the use of buccal dispersible antihistamines, pressurized aerosol bronchodilators, and oral corticosteroids.

3. Specific subcutaneous immunotherapy with 100% Apis mellifera extract.

4. Use of immunomodulatory drugs such as oral cyclosporine.

15- In a genetic disease with a recessive autosomal inheritance pattern, the risk of the disease in future offspring when both parents are carriers is:

1. 25% in each pregnancy regardless of the sex of the offspring.

2. 50% in each pregnancy for a male child.

3. 50% in each pregnancy for a female child.

4. 50% in each pregnancy regardless of the sex of the offspring.

16- In a certain gene, the DNA sequence is transcribed to form mature RNA, and this mature RNA is translated to form the protein. What part (sequence) of the structure of a gene is called an intron?

1. The one that starts the transcription to form the RNA.

2. The one that is not present in the mature RNA.

3. The one that is transcribed to be present in the mature RNA and give rise to the protein.

4. The one that regulates the expression of the gene.

17- To determine if the regular consumption of ultra-processed foods (e.g., sausages) is associated with a higher risk of frailty syndrome in older adults, 5,000 people over 70 years old living at home are selected. They are asked about their regular diet, and the consumption of ultra-processed foods is identified in each of them. A follow-up is conducted for 3 years to identify who develops frailty syndrome for the first time. Mark the correct answer about the design of this study:

1. It's a cohort study because subjects are followed based on their exposure to identify the risk of a health problem.

2. It's a field trial because it's conducted with people who aren't patients.

3. It's an ecological study because it includes a very large group of the population.

4. It's a case-control study where cases consume many ultra-processed foods, and controls consume very few.

18- Regarding systematic errors in observational studies, mark the INCORRECT statement:

1. They are classified into selection biases, information biases, and analysis biases.

2. They can be avoided by increasing the sample size.

3. They can modify the magnitude of the effect being investigated.

4. They can affect the internal validity of the study.

19- The negative predictive value of a diagnostic test is 92%. How should this result be interpreted?

1. Out of every 100 negative results, 92 will correspond to healthy individuals.

2. Out of every 100 healthy individuals, 92 will test negative.

3. Out of every 100 sick individuals, 8 will test negative.

4. Out of every 100 positive results, 8 will correspond to sick individuals.

20- The reason an intervention has a "strong" recommendation grade in a clinical practice guideline is:

1. It has more benefits than risks.

2. It's based on a high level of evidence.

3. It has a low cost.

4. It's preferred by patients.

21- In which of the following diseases should we apply contact and air (aerosol) isolation precautions?

1. Measles.

2. Tuberculosis.

3. Norovirus.

4. Chickenpox.

22- In the forest plot that displays the results of a systematic review of studies, the square next to each study represents:

1. The size of each study.

2. The estimator of interest (like relative risk) for each study.

3. The confidence interval for the estimator of interest in each study.

4. The weight of each study when conducting a meta-analysis of said studies.

Here are the translations of the given questions and answers:

24- The best preventive strategy to slow down the progression of the disease in a smoker diagnosed with COPD is to achieve the goal of quitting smoking. The following measure is NOT included in the current first-choice protocols for reinforcing smoking cessation treatment, because there are no demonstrative studies on its clinical efficacy and safety:

1. Nicotine patch.

2. Bupropion.

3. Varenicline.

4. Electronic cigarette.

25- Conjugate vaccines:

1. Are virus and bacteria vaccines combined to make them less immunogenic and cause fewer side effects.

2. Are whole virus vaccines conjugated with a polysaccharide to combine them and make the trivalent or tetravalent viral vaccine.

3. Are bacterial vaccines conjugated with a polysaccharide to combine them and create penta or hexavalent vaccines.

4. Are polysaccharide vaccines conjugated with a protein to make them T-cell dependent and achieve long-term immunological memory.

26- A clinical trial in which participants are randomly assigned to one of several clinical interventions is known as:

1. Double-blind trial.

2. Non-randomized controlled trial.

3. Randomized controlled trial.

4. Superiority trial.

27- In managing a pregnant woman diagnosed with bipolar disorder, which of the following drugs is effective in this disease and has been established as safer in pregnancy?:

1. Fluphenazine.

2. Olanzapine.

3. Lithium.

4. Carbamazepine.

28- A 69-year-old patient diagnosed with lymphoma is prescribed intravenous treatment with methotrexate. On the third day of treatment, he presents with nephrotoxicity and a delay in renal elimination of the drug with a urinary pH of less than 7. Given the chemical nature of methotrexate (pKa between 4 and 5.5), which of the following actions would be most appropriate to manage toxicity?:

1. Intravenous administration of physiological saline with ammonium chloride to facilitate renal elimination of methotrexate.

2. Intravenous administration of sodium bicarbonate solution to accelerate the diuresis of methotrexate.

3. Enteral administration of sodium bicarbonate solution to reduce digestive absorption of methotrexate.

4. Intravenous administration of furosemide in neutral pH saline to induce forced diuresis.

29- What is the mechanism by which ivabradine, acting on the cells of the sinoatrial node, produces a decrease in heart rate?:

1. Enhances repolarization by activating potassium outflow currents.

2. Modulates beta-1 adrenergic receptors.

3. Blocks the If pacemaker current.

4. Activates M2 muscarinic receptors.

30- In a patient with a duodenal ulcer and a high cardiovascular risk due to ischemic heart disease, if they require treatment with an NSAID, which of the following would you recommend as safer?:

1. Naproxen.

2. Celecoxib.

3. Ketorolac.

4. Ibuprofen.

31- A 68-year-old man with a history of obesity and type 2 diabetes mellitus, treated with metformin, is admitted for a first episode of heart failure. He has a glycosylated hemoglobin of 8.5%. To optimize diabetes treatment upon discharge, which of the following drugs is contraindicated?:

1. Linagliptin.

2. Canagliflozin.

3. Dulaglutide.

4. Pioglitazone.

32- A 5-year-old boy who had a bicycle accident with facial trauma without loss of consciousness, was only found in emergencies with a chin wound, which was sutured. He is referred to the clinic 5 days after the trauma due to limited and deviated mouth opening. What is the most likely diagnosis?:

1. LeFort fracture.

2. Combined fracture of the mandible and upper jaw.

3. Mandibular condyle fracture.

4. Symphysis and horizontal branch fracture of the mandible.

Here are the translations:

33- Of the following malignant tumors that can affect the tongue, which is the most common type and where does it usually metastasize?:

1. The sarcoma and it metastasizes in the cervical lymph nodes.

2. The squamous cell carcinoma and it metastasizes in the cervical lymph nodes.

3. The sarcoma and it metastasizes in distant bone tissue.

4. The squamous cell carcinoma and it metastasizes in hepatic tissue.

34- Which of the following descriptions defines stage II of a pressure ulcer according to the staging system of the National Pressure Injury Advisory Panel?:

1. Non-blanchable erythema.

2. Full-thickness skin loss with exposure of subcutaneous tissue.

3. Loss of epidermis with exposure of the dermis.

4. Appearance of granulation tissue in the defect bed.

35- Regarding skin grafts, mark the INCORRECT answer:

1. One of the beds considered graftable for its rich vascularization is granulation tissue.

2. We can classify them as partial skin grafts (if they only have the epidermis) or full skin grafts (if they have both the epidermis and the underlying dermis).

3. They usually undergo a contraction process, which appears both when they are extracted and after they have been grafted.

4. Among the medical comorbidities associated with graft loss, we can highlight diabetes, smoking, and peripheral vasculopathy.

36- A 38-year-old woman has been feeling a sensation of grit and burning in both eyes for several weeks, which worsens throughout the day, in windy conditions, and with air conditioning. Which of the following is the most likely diagnosis?:

1. Allergic conjunctivitis.

2. Episcleritis.

3. Pinguecula.

4. Dry keratoconjunctivitis.

37- Regarding posterior vitreous detachment, mark the INCORRECT statement:

1. The symptoms that patients usually consult for are phosphenes and floaters.

2. The Weiss ring is a characteristic sign in ophthalmoscopic examination.

3. The Shaffer sign or tobacco dust is a sign of good prognosis.

4. It is caused by the liquefaction of vitreous gel due to age.

38- A 25-year-old man has noticed a significant decrease in night vision and poor adaptation to darkness over the past few months. He is finding difficulty in driving, going downstairs, and lately has been tripping on curbs. However, he can read and watch television. Which of the following conditions is related to the symptoms of this patient?:

1. Stargardt's disease.

2. Best's vitelliform macular dystrophy.

3. Retinitis pigmentosa.

4. Retinal detachment.

39- A 22-month-old boy was treated for bilateral acute otitis media which presented with pain, fever, otorrhea, and hearing loss. Oral antibiotic treatment was initiated along with topical drops, which improved the symptoms. Twelve days later, he returns to the emergency room because the fever reappeared and the parents observed that the ear pinna has moved outwards and downwards, and the skin behind the ear is red and swollen. What is the most likely diagnosis?:

1. External otitis.

2. Mastoiditis.

3. Chondritis.

4. Cellulitis.

40- A 40-year-old woman presents with dizziness accompanied by nausea and vomiting, lasting about twenty minutes. She did not lose consciousness and was able to hold onto a wall to keep from falling. This has occurred before. She reports feeling ear fullness, tinnitus, and hearing loss in the right ear. Physical examination shows normal otoscopy, grade II left horizontal-rotatory nystagmus, right-sided positive Romberg, and Unterberger with rightward deviation. What is the most likely diagnosis?:

1. Benign paroxysmal positional vertigo.

2. Vestibular neuritis.

3. Ménière's disease.

4. Ramsay-Hunt syndrome.

41- Oro-pharyngeal dysphagia is the difficulty in moving food from the oral cavity to the proximal esophagus. The following are causes of motor oropharyngeal dysphagia EXCEPT:

1. Myasthenia gravis.

2. Gastroesophageal reflux.

3. Tetanus.

4. Dermatomyositis.

42- Regarding vulvovaginal infections, mark the INCORRECT statement:

1. Bacterial vaginosis is usually a mono-microbial infection.

2. Candida vulvovaginitis is the most prevalent vulvovaginal infection in Europe.

3. Recurrent candida vulvovaginitis is defined when there are 4 or more episodes in a year.

4. Bacterial vaginosis should only be treated pharmacologically when symptomatic.

43- Regarding the polycystic ovary syndrome, indicate the INCORRECT statement:

1. It occurs in young women.

2. Clinically it is characterized by the presence of hirsutism, acne, and amenorrhea or oligomenorrhea.

3. It is associated with obesity and insulin resistance.

4. It increases the risk of ovarian cancer.

44- A 35-year-old primigravida in the second stage of labor with epidural analgesia. When would the application of the vacuum to shorten the expulsive period be indicated?:

1. When, after 2 hours in full dilation, the baby's position remains at the second Hodge plane with maternal pushing.

2. When significant vaginal bleeding starts with fetal repercussions, and the baby's position is above the mother's pelvic inlet.

3. When the baby's position is at the fourth Hodge plane and the reference point is the chin.

4. When fetal bradycardia occurs that doesn't recover after contraction ends, and the posterior fontanel is past the third Hodge plane.

45- A 41-year-old pregnant woman at 13+2 weeks has a combined risk for chromosomal abnormalities for trisomy 21 of 1/30 after the 12-week ultrasound. What would be the most appropriate advice?:

1. Advance the 20-week ultrasound.

2. Perform an invasive test.

3. Reassure her because it's a combined low-risk in any case.

4. Reassure her because it's an intermediate risk and it's justified by maternal age.

46- A primigravida at 26 weeks of gestation, with normal monitoring to date, goes to her health center with an O'Sullivan test result of 175 mg/dL. In this situation, how should we proceed?:

1. She should be informed of the risk of gestational diabetes with fetal risk and referred to the hospital emergency department.

2. A 100g oral glucose tolerance test will be indicated only if she has risk factors for glucose metabolism disorders.

3. As it's a value above 165 mg/dL, the diagnosis of gestational diabetes is confirmed.

4. A diagnostic confirmation of gestational diabetes should be requested by performing a 100g oral glucose tolerance test.

47- A 36-year-old woman with a history of 2 first-trimester miscarriages and severe preeclampsia that required a cesarean section due to fetal distress at 33 weeks of gestation. The postpartum analytical study is normal except for the presence of antiphospholipid antibodies IgM and IgG, with the rest of the thrombophilia study being normal. The presence of antiphospholipid antibodies was confirmed 4 months after the first test. What recommendation would we give to this patient in case of a new pregnancy?:

1. Aspirin and low molecular weight heparin at prophylactic doses.

2. High-dose antioxidant vitamins.

3. Try pregnancy through preimplantation genetic diagnosis.

4. Treatment according to first-trimester preeclampsia screening with low-dose aspirin if high risk.

48- A 54-year-old nulliparous woman consults for urinary incontinence after feeling a urinary urgency and needing to urinate 3-4 times at night. During the gynecological examination, a first-degree cystocele and moderate vaginal atrophy are observed. What initial measures should be taken?:

1. Surgical correction of the cystocele.

2. Perform a urinary diary and bladder retraining.

3. Indication of uroflowmetry and urethral function evaluation.

4. Prescription of oral treatment with cholinergic drugs.

49- A 29-year-old primigravida without significant medical history is referred at 36.5 weeks of gestation due to suspected growth restriction by ultrasound. In the consultation, her blood pressure is 125/75 mmHg, and the obstetric ultrasound shows a fetal weight estimate compatible with the 6th percentile for gestational age and a Doppler pulsatility index of the umbilical artery above the 95th percentile. What recommendation would you make in this situation?:

1. Immediate termination of pregnancy through a cesarean section.

2. Weekly monitoring with fetal monitoring and obstetric ultrasound every two weeks until spontaneous labor begins.

3. Weekly monitoring with fetal monitoring and obstetric ultrasound every two weeks until 40 weeks of gestation.

4. Induction of labor from 37 weeks of gestation.

50- A 35-year-old woman consults for amenorrhea of 6 months' duration. In the medical history, she mentions recent difficulty sleeping and waking up at night feeling very hot. Menarche at 12 years old. She has 2 daughters (normal deliveries) and no further desire for children. Her mother entered menopause at 41 years. Blood tests show FSH 65.1 U/L (2-13.8), LH 12 U/L (2-13.8), estradiol < 0.05 pg/mL (follicular phase: 30-100; luteal phase: 50-150; ovulatory peak: 100-400). Two months later, similar results are observed in repeat tests. What is the suspected diagnosis and the approach to be taken?:

1. She's probably menopausal, but until 12 months of amenorrhea are completed, no treatment can be started.

2. Premature ovarian insufficiency. Start karyotype study and indicate hormone replacement therapy.

3. Premature ovarian insufficiency. Explain the importance of healthy lifestyle habits: no smoking, exercise, and adequate vitamin D intake. Hormone therapy is considered a second-line treatment.

4. Hypothalamic amenorrhea. Requires a cranial MRI study.

51- Which of the following vaccines should NOT be administered to a 9-month-old infant with primary combined immunodeficiency?:

1. 13-valent pneumococcal conjugate vaccine.

2. Influenza vaccine.

3. Rotavirus vaccine.

4. Type B meningococcal vaccine.

52- A 19-month-old boy, properly vaccinated for his age. Previously well, suddenly starts with high fever up to 40 ºC without other symptoms, except for irritability. The examination the next day is normal, except for slight redness of the oral mucosa and eardrum, and small lateral cervical lymph nodes. On the fourth day, the fever completely disappears and a reddish morbilliform, maculopapular rash appears, distributed on the face, trunk, and limbs. Among the following, what is the most probable diagnosis?:

1. Infectious erythema or fifth disease.

2. Measles or first disease.

3. Roseola, sudden exanthema, or sixth disease.

4. Scarlet fever or second disease.

53- An infant of 14 months presents to the emergency room with irritability over the last 12 hours and bloody stools with mucus. On examination, there are episodes of crying with flexion of the lower limbs. On abdominal palpation, an ill-defined cylindrical mass is observed, with the child's crying increasing when pressure is applied to the right side of the abdomen. What is your suspected diagnosis and what test would you perform to confirm it?:

1. Acute appendicitis. Abdominal ultrasound.

2. Intestinal intussusception. Abdominal ultrasound.

3. Acute appendicitis. Abdominal CT scan.

4. Intestinal intussusception. Abdominal CT scan.

54- Regarding acute gastroenteritis (AGE) in pediatrics, select the INCORRECT statement:

1. Rotaviruses are an infrequent cause of AGE in children worldwide.

2. The main bacterial pathogens causing AGE are non-typhoidal Salmonella, Shigella, Campylobacter, and Yersinia.

3. Clostridioides difficile disease can be both nosocomial and community-acquired in children.

4. Among the bacterial pathogens causing foodborne illnesses are Bacillus cereus, Clostridium perfringens, and Staphylococcus aureus.

55- Among the following clinical characteristics related to anorexia nervosa, select the correct statement:

1. One clinical form is associated with binge eating and purgative behaviors.

2. It is distributed equally among both sexes.

3. Amenorrhea is a necessary diagnostic criterion for the disease in women.

4. There's a desire to lose weight but with a normal perception of one's own constitution.

56- Concerning hypertrophic pyloric stenosis, select the INCORRECT statement:

1. It predominantly affects first-born males.

2. A higher incidence has been found in daughters of mothers treated with macrolides during pregnancy.

3. The typical hydroelectrolytic disturbance is a hypochloremic metabolic acidosis.

4. The treatment of choice is Ramstedt's extramucosal pyloromyotomy.

57- For the diagnosis of classic Kawasaki disease, the presence of fever for at least five days and 4 of the main symptoms of the disease are required. Of the following, indicate which is NOT CONSIDERED a main sign:

1. Erythema of the pharyngeal and oral mucosa with strawberry tongue and/or cracked lips.

2. Polymorphic rash.

3. Cervical lymphadenopathy (> 1.5 cm in diameter), generally unilateral.

4. Bilateral exudative conjunctival injection.

58- Concerning vaccines and vaccination schedules in the autonomous communities of Spain, select the INCORRECT statement:

1. Systematic vaccines are those that are mandatorily administered to all children according to the current vaccination schedule in their autonomous community.

2. Non-systematic vaccines are those that are not administered universally in all autonomous communities.

3. The vaccination schedule is defined as the chronological sequence of vaccines that are systematically administered in a particular country or geographic area.

4. Vaccination schedules should be permanently updated based on the development of new vaccines or changes in the incidence of diseases in a particular geographic area.

59- A 7-year-old patient has been hospitalized for several days with a diagnosis of pneumonia and has been treated with antibiotics. He continues to have fever spikes, so a thoracic ultrasound is performed, revealing pleural effusion with multiple septa. A diagnostic thoracocentesis is performed, extracting purulent material. What should be done next?:

1. Closed drainage with a chest tube with instillation of fibrinolytics.

2. Repeated thoracocenteses to remove all possible effusion.

3. Surgical decortication.

4. Broadening of the antibiotic coverage.

60- A 13-year-old female presents to the clinic with high fever for 5 days, odynophagia, headache, abdominal pain, and severe fatigue that interferes with her daily life. On examination, red tonsils with an exudative membrane are seen, which are foul-smelling. Anterior, posterior, occipital, and epitrochlear cervical lymph nodes are palpable. The liver is palpable 1 cm and the spleen 3 cm from the costal margin. Given these symptoms and examination findings, which serological test would you request?:

1. Measles.

2. Rubella.

3. Epstein-Barr.

4. Parvovirus B 19.

61- Indicate which drug is NOT indicated in the maintenance treatment of bipolar disorder:

1. Lithium.

2. Valproate.

3. Carbamazepine.

4. Clorazepate.

62- A 26 -year-old male has had a first depressive episode with complete remission of symptoms with a first monotherapy antidepressant. How long is it recommended to continue the treatment?:

1. In a first episode in young patients with a good response, treatment can be discontinued after a month.

2. It is recommended to maintain the treatment for a minimum of three months after remission.

3. It is recommended to maintain the treatment for a minimum of six months after remission.

4. It is recommended to maintain the treatment for a minimum of two years to prevent relapses.

Here are the English translations for the given text:

63- A 71-year-old male without psychiatric history presents with a three-week-long condition characterized by memory deficit, inattention, insomnia, weight loss, restlessness, morning-predominant anguish, and delusional ideas of guilt and harm. From the following, the most probable diagnosis is:

1. Catalepsy.

2. Late-onset schizophrenia.

3. Alzheimer's disease.

4. Severe depressive episode with psychotic symptoms.

64- Major depression, especially if it has an endogenous/melancholic character, is characterized by one of the following alterations:

1. Increased appetite.

2. Decrease in cortisol secretion.

3. Difficulty waking up in the morning.

4. Worsening in the morning.

65- Indicate which of the following disorders is NOT an anxiety disorder according to the DSM-5:

1. Obsessive-compulsive disorder.

2. Selective mutism.

3. Situational phobia.

4. Panic disorder.

66- In psychogeriatrics, when the use of benzodiazepines is necessary, which is the drug of choice?:

1. Clorazepate.

2. Lorazepam.

3. Diazepam.

4. Benzodiazepines are contraindicated.

67- Regarding the side effects of antipsychotics, identify the INCORRECT statement:

1. The appearance of metabolic syndrome is more common with the use of typical antipsychotics.

2. Sedation is a common side effect with most antipsychotics, more pronounced at the beginning of treatment.

3. Tardive dyskinesia is an involuntary movement disorder that most commonly affects the orofacial region, appears after prolonged administration of antipsychotics, and can be irreversible.

4. Akathisia is an extrapyramidal effect for which the addition of benzodiazepines or the use of beta-blockers may be indicated.

68- Regarding the combined therapy with psychotropic drugs and psychotherapy for mental disorders, it's true that:

1. It's only indicated when both forms of treatment have failed separately.

2. The rapid relief of anxiety symptoms thanks to medication decreases motivation for psychotherapy.

3. It requires the same professional to carry out the two treatment modalities.

4. The results of combined therapy are superior to those of each used separately.

69- Within the primary parkinsonian syndromes, Parkinson's disease occurs due to:

1. A neuronal degeneration that especially affects the globus pallidus, reticular substance, and inferior olive, producing a dopamine deficit.

2. Degeneration of the nuclei of the protuberance, inferior olive, and cerebellum.

3. Cellular degeneration in the substantia nigra that reduces the dopamine rate at the striatal level, causing excessive subthalamic and pallidal activity.

4. Progressive neuronal degeneration at the cortical and basal ganglia levels.

70- Regarding the neurological examination of a lesion in the corticospinal tract, identify the INCORRECT statement:

1. There is global weakness in all muscle groups of the affected limbs.

2. The Babinski reflex is present.

3. It presents rigidity-type hypertonia.

4. There is hyperreflexia in the patellar tendon reflexes.

71- A 52-year-old male, diabetic, consults for painless right foot drop. On examination, there is weakness of the right anterior tibial muscle and peroneals, with preserved patellar and Achilles reflexes. What is the most likely diagnosis?:

1. Right S1 radiculopathy.

2. Right sciatic popliteal neuropathy.

3. Right L5 radiculopathy.

4. Diabetic amyotrophy.

72- In a patient with a two-year parkinsonian syndrome evolution, identify which of the following clinical manifestations would make you doubt a diagnosis of Parkinson's disease:

1. Severe dysarthria.

2. Absence of tremor.

3. Presence of a REM sleep behavior disorder.

4. Constipation.

73- A 19-year-old student suffers his first generalized tonic-clonic seizure while lying on the sofa. Neurological examination is normal. In a directed anamnesis, he mentions that he had been sleeping fewer hours in recent days because he was studying for exams, and that sometimes in the mornings, he experiences jerks in his arms when grabbing his breakfast mug. His EEG shows generalized spike and wave discharges. Indicate the INCORRECT answer:

1. The cranial MRI is probably normal.

2. I would not start treatment as this is his first seizure.

3. The most likely diagnosis is juvenile myoclonic epilepsy.

4. I would start treatment with valproate.

74- A 21-year-old male begins to show psychiatric symptoms including hallucinations, behavioral disturbances, and episodes of catatonia. After a few days, he starts to experience memory deficits and oral dyskinesia, and on the tenth day, he begins having seizures. Which of the following tests would provide the most information for the diagnosis?:

1. Brain MRI with spectroscopy.

2. Long-duration EEG with video-EEG.

3. Initiate a treatment cycle with high doses of methylprednisolone to assess response.

4. Study in CSF and serum for specific autoimmune antibodies.

75- A 53-year-old woman without significant medical history goes to the emergency room due to a sudden speech impairment which started an hour ago. The only noticeable symptom is motor aphasia. The baseline cranial CT scan is normal. The perfusion CT shows a flow defect in the left frontal cortical region without any volume map impairment. The CT angiogram reveals a clot in the distal segment (M3-M4) of the left middle cerebral artery. Indicate the most appropriate treatment:

1. Mechanical thrombectomy.

2. Intravenous fibrinolysis with rTPA.

3. Intravenous fibrinolysis and, if recanalization is not achieved, mechanical thrombectomy.

4. Anticoagulation with sodium heparin.

76- In patients with tuberous sclerosis (Bourneville's disease), indicate the INCORRECT answer:

1. They often have epileptic seizures.

2. They often have facial angiofibromas.

3. The most common brain tumor is the subependymal giant cell astrocytoma.

4. They often have neurinomas.

77- A 64-year-old woman presents with facial pain in the territory of the third branch of the fifth cranial nerve (trigeminal nerve). Regarding the diagnosis of trigeminal neuralgia, indicate the INCORRECT statement:

1. Excruciating pain of very short duration.

2. The pain has a trigger point, associated with tactile stimulation of the affected area.

3. The pain can spontaneously disappear after persisting for weeks or months.

4. It is associated with facial hypoesthesia.

78- Regarding post-traumatic epileptic seizures, it's true that:

1. They are common immediately after the impact.

2. Penetrating injuries and the severity of the injury are favoring factors.

3. Prophylactic anticonvulsant treatment is indicated.

4. After a couple of years of increased risk, patients with severe injuries have the same risk as the general population.

79- A 25-year-old male is admitted to the ICU after an undetermined drug overdose. Due to a progressive decrease in consciousness level, which doesn't improve with naloxone or flumazenil, intubation and invasive mechanical ventilation are decided. Which of the following drugs would NOT be used for orotracheal intubation?:

1. Quetiapine.

2. Rocuronium.

3. Etomidate.

4. Fentanyl.

80- A 50-year-old man with Legionella pneumonia requires ICU admission due to acute respiratory failure. He needs to be intubated and connected to invasive mechanical ventilation. He's diagnosed with acute respiratory distress syndrome and is placed in the prone position. Regarding this positional technique, indicate the INCORRECT statement:

1. It is done to try to increase the ratio between the partial pressure of oxygen and the fraction of inspired oxygen.

2. It is carried out by trained personnel to prevent complications.

3. The ratio between the partial pressure of oxygen and the fraction of inspired oxygen below which it is recommended is 300 mmHg.

4. The prone position during invasive mechanical ventilation requires deep sedation.

81- A 58-year-old patient suffers from COVID-19 pneumonia and requires intubation with mechanical ventilation in the ICU. A week later, a tracheostomy is performed, but the patient still requires mechanical ventilation. A motor deficit is detected in all four limbs with generalized hyporeflexia. The initial diagnostic orientation and therapeutic attitude to follow are:

1. Guillain-Barré Syndrome. Confirm the diagnosis with complementary tests.

2. Post-COVID Syndrome. Respiratory rehabilitation.

3. Critical illness myopathy. Corticosteroids and complementary tests.

4. Critical illness myopathy. Intensive respiratory and motor rehabilitation.

82- A 55-year-old man with no significant medical history feels lumbar pain radiating to the right lower limb on the anterolateral part of the thigh and anterior side of the knee after exerting himself. Examination: positive Lasègue sign at 40º, diminished patellar reflex, and difficulty walking on heels. What is the most likely diagnosis?:

1. L2-L3 right disc herniation.

2. L3-L4 right disc herniation.

3. L4-L5 right disc herniation.

4. L5-S1 right disc herniation.

83- A 5-year-old patient comes to the emergency room with a fever that has lasted for 48 hours, responding poorly to antipyretics and refusing to put weight on the left lower limb. Upon physical examination, limited mobility of the hip is observed. The blood test shows an increased CRP (C-reactive protein) and leukocytosis. Which complementary test should be requested first?:

1. Hip X-ray.

2. Hip ultrasound.

3. MRI of both hips.

4. Hip arthrography.

84- An 82-year-old woman with a history of congestive heart failure and breast cancer consults due to a left wrist fracture. The fracture is stable, not comminuted, and does not present intra-articular lines. Which of the following treatments is most appropriate?:

1. Closed reduction and forearm cast.

2. Total wrist arthroplasty.

3. Osteosynthesis with dorsal and volar plates.

4. Do not immobilize, immediate rehabilitation.

85- A 35-year-old housekeeper, with no significant medical history, right-handed, reports pain in the 5th finger of the left hand and on the inner side of the left forearm, accompanied by numbness and partial loss of sensitivity. What would be the first diagnostic suspicion?:

1. Carpal tunnel syndrome.

2. C3-C4 cervical disc herniation.

3. Cubital tunnel syndrome at the elbow.

4. De Quervain's tendinitis.

86- Regarding the nonunion of the scaphoid, indicate the INCORRECT statement:

1. It usually results in necrosis of the distal fragment of the fracture.

2. It can be asymptomatic.

3. In the long term, it usually causes radiocarpal arthritis.

4. In the early stages, the treatment of choice is fracture fixation and bone grafting.

87- A 69-year-old man, with a BMI of 37 and significant central (abdominal) obesity, suffers from debilitating lower back pain that extends to both buttock regions but does not radiate to the lower limbs. It prevents him from standing still and walking more than 10 minutes, but disappears when sitting and in bed. The Lasègue test is negative. What is the most likely diagnosis?:

1. L5-S1 disc herniation.

2. Lumbar canal stenosis.

3. L4-L5 disc herniation.

4. Bacterial spondylodiscitis.

88- In the face of a lytic diaphyseal lesion of the humerus affecting the entire diameter of the bone in a 67-year-old man with a history of lung cancer (expected survival of more than 2 years) causing continuous poorly controlled pain, which of the following is the best therapeutic approach?:

1. Wait and see approach, as it is a non-weight bearing bone, reducing fracture risk.

2. Application of high-dose opioids to control the pain and allow physiotherapy to restore function.

3. Properly apply a cast to reduce fracture risk.

4. Placement of a locked intramedullary nail.

89- A 29-year-old man comes in with lower back pain, swelling of the hands that has lasted several weeks, along with blurry vision and red eyes. He's had multiple unprotected sexual encounters and there are cases of psoriasis in his family. Physical examination shows a temperature of 37.5°C, bilateral conjunctival injection, and swelling of the distal parts of the fingers and the left wrist, with positive sacroiliac tests. What is the most likely diagnosis?:

1. Reactive arthritis.

2. Psoriatic arthritis.

3. Enteropathic arthritis.

4. Ankylosing spondylitis.

90- A 55-year-old man with a history of hyperuricemia comes to the emergency room with a first episode of very intense and debilitating pain in the right knee, accompanied by redness, swelling, and puffiness. Mild fever of 37.5°C. What is the most recommended approach?:

1. Perform arthrocentesis and send the synovial fluid to the laboratory to check for the presence of crystals and germs.

2. Start treatment with colchicine because he is experiencing a gout attack.

3. Start treatment with an NSAID and allopurinol to treat the gout attack and hyperuricemia.

4. Start treatment with an NSAID, allopurinol, and an antibiotic to treat the gout attack, hyperuricemia, and the probable infection.

91- An 81-year-old asthmatic woman who frequently uses corticosteroids to control bronchospasm flare-ups consults because she has intense sharp pain in the dorsolumbar region after falling at home from a chair. What is the most recommended approach?:

1. Urgently request dorsal and lumbar spine X-rays and prescribe pain relief treatment.

2. Request an MRI of the dorsal and lumbar spine and prescribe pain relief and rest.

3. Prescribe pain relief and start anti-resorptive treatment.

4. Request a bone density test, prescribe pain relief, and start anti-resorptive treatment, calcium, and vitamin D.

92- Which of the following is NOT a criterion for the diagnosis of infectious endocarditis?:

1. Presence of major arterial embolic events.

2. A single positive blood culture for Coxiella burnetii.

3. At least two positive blood cultures taken >12 hours apart for microorganisms consistent with infectious endocarditis.

4. Presence of hemolytic anemia.

93- Which of the following criteria is considered high risk in the stratification of syncope?:

1. Syncope caused by turning the head or pressing on the carotid sinus.

2. Syncope during exertion or in the supine position.

3. Syncope after seeing, hearing, or smelling something unpleasant.

4. Second-degree Mobitz I atrioventricular block.

94- Indicate which of the following tachycardias is NOT explained by a reentry mechanism:

1. Atrial flutter or flapping.

2. Polymorphic ventricular tachycardia type torsade de pointes.

3. Reciprocal atrioventricular tachycardia in Wolff-Parkinson-White syndrome.

4. Tachycardias associated with defects in the interatrial septum.

95- A 68-year-old male with a mechanical mitral valve, anticoagulated with acenocumarol, presents an INR of 9 in a routine check-up at his health center. He has no current bleeding symptoms. Which of the following is the most correct approach?:

1. Discontinue acenocumarol treatment and administer 10 mg of vitamin K in slow intravenous infusion, repeating every 12 hours if necessary.

2. Stop acenocumarol and refer the patient to a hospital center to monitor for bleeding.

3. Temporarily discontinue acenocumarol and resume anticoagulation when the therapeutic interval has been reached, recalculating the dose.

4. Start administration of prothrombin complex concentrate or fresh frozen plasma based on weight.

96- Regarding cardiac tamponade, which of the following statements is true?:

1. Echocardiographic manifestations and hemodynamic changes occur before clinical manifestations appear.

2. Hypotension, tachycardia, decreased jugular venous pressure, and attenuated cardiac sounds are typical.

3. The volume of pericardial fluid required to produce tamponade is similar in acute pericarditis and chronic pericarditis.

4. Paradoxical pulse is a characteristic physical examination finding defined as a drop of 5-10 mmHg in systolic blood pressure during inspiration.

97- A 71-year-old male, diabetic, with no other comorbidities, previously diagnosed with severe aortic stenosis. Admitted for an acute coronary syndrome without ST elevation, Killip I with a slightly elevated troponin peak. The catheterization shows coronary artery disease with significant proximal lesions in the three coronary arteries. His ventricular function is normal. Which of the following therapeutic strategies is the most correct?:

1. Interventional treatment with the implantation of coronary stents in the 3 vessels and simultaneous implantation of a transcatheter aortic bioprosthesis (TAVI).

2. Surgical mechanical aortic valve prosthesis, followed by percutaneous coronary interventional treatment at a later time.

3. Transcatheter biological aortic prosthesis (TAVI) and then, on an outpatient basis, percutaneously treat the coronary arteries with stents.

4. Simultaneous surgical treatment with valve replacement and coronary bypass surgical revascularization.

98- A 75-year-old male reports progressive dyspnea, with decreasing exercise tolerance. He reports syncope on two occasions in the past few months, coinciding with moderate effort, and occasionally reports chest pain that increases with effort. On examination, the presence of a parvus et tardus arterial pulse and a mesosystolic murmur stand out. The blood test is normal except for a creatinine of 2.2 mg/dL. Surgical risk assessment establishes an intermediate risk. Indicate which of the following therapeutic options is most appropriate:

1. Surgical replacement of the mitral valve.

2. Percutaneous implantation of the aortic valve (TAVI).

3. Surgical replacement of the aortic valve.

4. Percutaneous mitral valvulotomy.

99- A 43-year-old woman, a professional swimmer, with no relevant medical history, reports swelling and heaviness of her right arm, lasting 72 hours. On examination, there is edema of the right upper limb with the presence of dilated veins in the pectoral region. Regarding the diagnostic approach, indicate the INCORRECT answer:

1. Color Doppler ultrasound is of great value as the first diagnostic test.

2. A simple chest x-ray should be performed to detect bone abnormalities.

3. Angio-CT confirms the diagnosis.

4. A phlebography should be performed to confirm the presence of axillo-subclavian venous thrombosis.

100- A 72-year-old male comes to the emergency room with fever, cough, and purulent sputum that has been present for three days. On examination, he is oriented with a good level of consciousness. Blood pressure is 110/75 mmHg, respiratory rate is 32 breaths/min. Lung auscultation reveals crackles in the right mid-lung field. Chest x-ray shows an alveolar infiltrate in the middle lobe. Indicate the correct statement about the severity criteria listed in the CRB-65 scale and the course of action to take:

1. Score of 0 points, and he would be eligible for discharge and outpatient treatment.

2. Score of 1 point, and he would be eligible for discharge and outpatient treatment depending on the doctor's judgment.

3. Score of 2 points, and he may require hospital admission depending on the doctor's judgment.

4. Score of 3 points, and requires hospital admission.

101- A 67-year-old male diagnosed with COPD with a GOLD 1 category, smoker, reports dyspnea with intense efforts, without exacerbations. Which of the following is the most correct therapeutic recommendation?:

1. Regular treatment with long-acting muscarinic bronchodilators (LAMA).

2. Regular treatment with long-acting beta-adrenergic bronchodilators (LABA).

3. Treatment with LABA plus inhaled glucocorticoids.

4. Non-pharmacological treatment.

102- A 70-year-old woman admitted for severe traumatic brain injury with secondary subarachnoid hemorrhage. On the second day of admission, she experiences dyspnea and chest pain. Chest angioCT confirms the diagnosis of pulmonary embolism. The treatment of choice is:

1. Low molecular weight heparin.

2. Rivaroxaban.

3. Acenocumarol.

4. Inferior vena cava filter.

103- A 71-year-old patient presents with jaundice showing a total bilirubin of 6 mg/dL (direct bilirubin 4 mg/dL), normal transaminases, and elevated GGT at 349 U/L and alkaline phosphatase at 260 U/L. An abdominal ultrasound reveals dilation of the intrahepatic and extrahepatic bile ducts. The patient reports no pain but mentions a weight loss of about 5 kg in the last month. A retrograde cholangiopancreatography is performed, which shows a sharp stenosis of the distal common bile duct. What is your primary diagnostic suspicion?

1. Primary sclerosing cholangitis.

2. Choledocholithiasis.

3. Pancreatic cancer.

4. Acute hepatitis.

104- A 58-year-old man with compensated cirrhosis Child A-5 due to non-alcoholic fatty liver disease with no other history. He reports fatigue without anorexia or weight loss. A screening abdominal ultrasound and a subsequent triphasic thoracoabdominal CT scan detect four hepatic lesions (one of them 6 cm in size), hypervascular in the arterial phase with venous phase washout, with portal vein invasion and no extrahepatic metastases. No ascites is observed. Which of the following is the best treatment?

1. Transarterial chemoembolization.

2. Sorafenib.

3. Liver transplant.

4. Radiofrequency ablation.

105- Which of the following types of acute appendicitis most frequently presents with a positive psoas sign?

1. Pelvic appendicitis.

2. Mesoceliac appendicitis.

3. Retrocecal appendicitis.

4. Medioinguinal appendicitis.

106- A 45-year-old patient had an episode of cholecystitis three months ago, where gallstones were found on ultrasound. Currently, they present with elevated alkaline phosphatase, and a new ultrasound shows small-sized gallstones with a bile duct measuring 1 cm in diameter. Which of the following investigations would you perform before surgical intervention?

1. Retrograde cholangiography.

2. Endoscopic ultrasound.

3. Transparietohepatic cholangiography.

4. Magnetic resonance cholangiography.

107- Regarding anal fissures, indicate the INCORRECT statement:

1. It is a longitudinal tear in the skin covering the portion of the anal canal below the dentate line, causing intense pain during defecation.

2. There are two typical forms: primary or idiopathic which appears in healthy individuals and isn't associated with any disease; secondary is found in patients suffering from another condition.

3. Most primary fissures are located in the anterior commissure, as this is an area of the anal canal more susceptible to trauma and mucosal ischemia.

4. Fissures secondary to other diseases often appear in more lateral positions.

108- A 24-year-old woman underwent surgery a year ago for endometriosis after experiencing episodes of intense abdominal pain, especially related to the menstrual cycle, occasionally accompanied by nausea and vomiting. Despite the surgery, she has not noticed any improvement, with progressively worsening pain spreading throughout her body, accompanied by limb weakness. Routine tests are normal, antinuclear antibodies and rheumatoid factor are negative, only some isolated tests show a Na of 132 mEq/L and slightly elevated ALT (GOT). Recent abdominal ultrasound and abdominal-pelvic CT scan are normal. Two months ago she was diagnosed with depression and fibromyalgia. Antidepressant and analgesic treatment with opioids doesn't seem to have any effect. She has been bedridden for months. We are asked for a second opinion. Indicate which of the following diagnostic tests should be checked if it has been performed:

1. MEFV gene study.

2. Porphobilinogen and aminolevulinic acid in urine.

3. Mitochondrial antibodies in blood.

4. Calprotectin in stool.

109- A 34-year-old man presents to the emergency room with dyspnea evolving over three weeks, along with intermittent red urine in the last ten days. Physical examination reveals: temperature 36.6ºC, BP 132/72 mmHg, respiratory rate 16 rpm, and heart rate 88 bpm, with fine scattered crackles on lung auscultation. Blood test: BUN 56 mg/dL, creatinine 5.8 mg/dL. Urine test: red blood cells 4+, proteins 2+. A renal biopsy reveals diffuse glomerulonephritis with crescents. Which of the following tests would confirm the diagnosis?:

1. Complement C3 concentration.

2. Titers of anti-glomerular basement membrane antibodies.

3. Anti-streptolysin O titers.

4. Stool culture for Escherichia coli O157:H7.

110- A 36-year-old patient presents to the emergency department in poor general condition. No prior tests available. Creatinine is detected at 10 mg/dL, Hb 6.5 g/L, absence of microalbuminuria, PTH 500 pg/ml, and normal total diuresis. Ultrasound shows reduced kidney size. Which of the following diagnoses is most likely?:

1. Autosomal dominant polycystic kidney disease.

2. Rapidly progressive glomerulonephritis.

3. Chronic tubulointerstitial nephropathy.

4. Diabetic nephropathy.

111- A 73-year-old man presents to the emergency room with pain in the lumbosacral area radiating to both flanks and hypogastrium evolving over two weeks that doesn't relieve with high doses of ibuprofen. He has a history of hypertension, infrarenal aortic aneurysm, and L5-S1 disc herniation. He reports a weight loss of 6 kg in the last month. Examination: Fair general condition, mucosal pallor, and doubtful bilateral renal fist percussion, no other findings. Blood test: Hb 8.2 g/dL, MCV 81 fL, leukocytes 6,330 (normal formula), glucose 89 mg/dL, urea 228 mg/dL, creatinine 5.4 mg/dL, Na 134 mEq/L, K 5.1mEq/L, Ca 11.3 mg/dL, total proteins 8.2 g/dL, albumin 3.0 g/dL, CRP 0.54 mg/dL. Urine: proteins 30 mg/dL with estimated proteinuria 1.5 g/24h. Urine sediment: leukocytes 20-30/field, erythrocytes >50/field. What is the most likely diagnosis causing kidney failure?:

1. Acute interstitial nephropathy due to analgesics.

2. Multiple myeloma.

3. Acute pyelonephritis.

4. Progression of the aortic aneurysm with bilateral involvement of the renal arteries.

112- A 45-year-old man is brought to the emergency department after having tonic-clonic seizures in public. He is confused and gives incoherent and vague answers. Reviewing his history, he is diagnosed with schizophrenia and depression, but he seems to have been off his medication for weeks. Examination: Stuporous, with no other findings. Blood tests with normal hemogram, glucose 98 mg/dL, urea 37 mg/dL, creatinine 0.8 mg/dL, Na 118 mEq/L, K 3.4 mEq/L, Ca 9.7 mg/dL, serum osmolarity 252 mOsm/kg, urinary osmolarity 78 mOsm/kg, urine density 1002, normal sediment. What is the most likely cause of his electrolyte disturbances?:

1. Adrenal insufficiency.

2. Primary polydipsia.

3. Diabetes insipidus.

4. Syndrome of inappropriate ADH secretion.

113- Regarding radical prostatectomy for the treatment of prostate cancer, select the INCORRECT answer:

1. Factors related to continence after a radical prostatectomy include advanced age, urethra length, and surgeon experience.

2. Recovery of erectile function after radical prostatectomy is associated with younger individuals, quality of erections before surgery, and no damage to neurovascular bundles.

3. Alprostadil can help restore satisfactory sexual function.

4. A PSA >0.01 after a radical prostatectomy indicates biochemical recurrence.

114- It's true in prostate cancer that:

1. Patients with clinically localized disease are treated with radical prostatectomy, radiotherapy, or active surveillance.

2. For its diagnosis, contemporary schemes recommend biopsies with 10 cores.

3. After radical prostatectomy, PSA becomes undetectable in blood in two weeks.

4. In its treatment, first-generation non-steroidal antiandrogens are used, such as bicalutamide and abiraterone.

115- Performing selective sentinel lymph node biopsy and lymphatic mapping in breast cancer:

1. Is indicated in disseminated breast cancer.

2. Is indicated if there is clinical involvement of the axillary lymph nodes.

3. If negative, there's no need to expand axillary surgery.

4. Is not indicated in males with breast cancer.

116- Regarding treatment with radiotherapy, indicate the INCORRECT answer:

1. It can be an alternative curative treatment in various situations in oncology.

2. It is also used for certain benign pathologies.

3. It's contraindicated for the treatment of pediatric tumors.

4. It has proven utility in controlling pain from bone metastases.

117- Patient diagnosed with glioblastoma multiforme. The pathological anatomy report mentions the presence of methylation of the MGMT gene promoter (methyl guanine methyl transferase). Regarding this patient's treatment:

1. This information is not relevant.

2. The presence of methylation increases survival in patients treated with temozolomide.

3. The presence of methylation would contraindicate treatment with temozolomide.

4. The presence of methylation would support the decision to treat with cisplatin over the use of an alkylating agent.

118- Radiotherapy in the treatment of locally advanced breast cancer with an adverse molecular prognosis:

1. Should include locoregional lymph node regions.

2. Is contraindicated after mastectomy.

3. Is contraindicated after neoadjuvant chemotherapy.

4. Its best aesthetic alternative is partial breast irradiation.

119- 19-year-old male, asymptomatic, who shows thrombocytopenia (platelets 43 x 109/L) with normal hemoglobin and leukocyte levels in a preoperative analysis. Which of the following actions should be taken first?:

1. Start treatment with oral prednisone.

2. Perform a bone marrow biopsy to determine if the thrombocytopenia is of central or peripheral origin.

3. Perform a morphological examination of the blood smear to rule out the presence of platelet aggregates induced by the anticoagulant.

4. Transfuse a platelet concentrate to prevent a bleeding problem.

120- 35-year-old male, diagnosed with hemophilia A since childhood, who receives a liver transplant due to chronic hepatitis C liver disease. Six months after the procedure, he presents a normal coagulation study with a factor VIII activity of 100%. Which of the following statements is true?:

1. Hemophilia has been cured since factor VIII is synthesized in the liver.

2. Immunosuppressive treatment has solved the problem as we are facing an autoimmune condition.

3. The long half-life of the antihemophilic factors used in the transplant means their effect lasts several months.

4. It can only be an error or laboratory artifact, as hemophilia A is an incurable genetic disease today.

121- 65-year-old male who consults for progressive fatigue and tingling sensation in hands and feet. Physical examination shows a yellowish complexion and a decrease in vibratory and positional sensitivity in distal areas. In the blood count, MCV is 120 fl with anisocytosis. It is most likely that he suffers from:

1. Cervical spondylosis myelopathy.

2. Subacute necrotizing myelitis.

3. Funicular myelosis.

4. Chronic myelopathy of multiple sclerosis.

122- 41-year-old male admitted to the ICU for severe pneumonia requiring intubation and administration of catecholamines. On examination, there's bleeding around the venipuncture sites. Laboratory results are: 15,500 leukocytes; Hb 10.4 g/dL; Hct 32%; 52,000 platelets; LDH 820 U (normal 110-210); reticulocyte count 4%. Which of the following results is NOT expected in this patient?:

1. Low fibrinogen.

2. High concentrations of antithrombin and protein C.

3. Prolonged prothrombin time and activated partial thromboplastin time.

4. Presence of fibrin degradation products (D-dimer).

123- Which of the following factors has NOT specifically been associated with a higher risk of falls in older people?:

1. Osteoporosis.

2. Visual impairment.

3. Osteoarthritis.

4. Polypharmacy.

124- The Short Physical Performance Battery (SPPB) is a physical performance test used to assess physical function, frailty, and fall risk in the elderly. What aspects does this test evaluate?:

1. Balance, walking speed, and the ability to climb up and down stairs.

2. Balance, walking speed, and the ability to rise from and sit down on a chair.

3. Balance, walking speed, and upper limb strength.

4. Balance, the ability to climb up and down stairs, and the ability to rise from and sit down on a chair.

125- One of the following mechanisms has NOT been proposed in the basic pathogenesis of delirium in the elderly patient:

1. Neuroinflammation.

2. Oxidative stress.

3. Excessive cholinergic activity.

4. Excess dopamine.

126- Regarding potentially inappropriate prescriptions in the elderly, it is true that:

1. Benzodiazepines do not increase the risk of falls.

2. There are no criteria to facilitate the correct prescription of drugs in this population group.

3. The use of metformin in diabetics with glomerular filtration rates below 30 ml/min is recommended.

4. Topical vaginal estrogens or estrogen pessaries are indicated for symptomatic atrophic vaginitis.

127- In relation to the functional assessment of the elderly patient, indicate the INCORRECT answer:

1. It should be interpreted as a global measure of the overall impact of health problems in the elderly patient.

2. There is a mandatory progressive hierarchy for both the acquisition and the loss of activities of daily living (ADL), from instrumental to advanced.

3. In hospitalized elderly patients, the functional status prior to hospital admission should be used to establish the treatment plan and set realistic intervention goals.

4. It is usually measured with self-reported or third-party reports, although sometimes, some team members perform a structured assessment to measure actual functional capacity.

128- 79-year-old woman with a history of hypertension and bilateral knee osteoarthritis, independent for daily living activities, lives alone. During a visit to the family doctor, sarcopenia is assessed. She completes the chair rise test for 5 lifts in 20 seconds, and the Timed Up and Go test in 26 seconds. Indicate the correct answer:

1. She is not at risk of falling.

2. She has a confirmed diagnosis of sarcopenia.

3. If she had sarcopenia, it would be severe.

4. Sarcopenia is not suspected.

129- 24-year-old male with bilateral gynecomastia, sparse body hair, 3 cc firm testes, total testosterone 257 ng/dL (N>350), LH 11 mUI/mL (N<10), FSH 24 mUI/mL (N<10). The next step in the diagnosis is:

1. Karyotype.

2. Testicular biopsy.

3. Pituitary MRI.

4. FSH receptor study.

130- Regarding hyperprolactinemia, indicate the INCORRECT statement:

1. Hyperprolactinemia can be caused by drugs.

2. Hyperthyroidism causes an elevation of prolactin.

3. The medical treatment of choice is dopaminergic agonists.

4. It causes hypogonadotropic amenorrhea.

131- 63-year-old woman with type 2 diabetes mellitus for seven years, treated with metformin. HbA1c 7.6%, body mass index 41.3 kg/m2. For the treatment of obesity in this patient, which is the best option among the following?:

1. Pioglitazone.

2. Empagliflozin.

3. Semaglutide.

4. Alogliptin.

132- 24-year-old woman consulting for severe sore throat lasting two days. She mentions she had muscle pain and dry cough about ten days ago, which improved last week. She feels shaky and sweaty. Examination shows no redness or pharyngeal exudate, but there is pain on palpation in the lower anterior cervical region, with no nodules, masses, or lymph nodes. Physical examination reveals a pulse of 110 bpm, temperature of 38.2ºC, and a slight tremor at rest. What is the most likely diagnosis?:

1. Subacute thyroiditis.

2. Focal lymphocytic thyroiditis.

3. Acute or infectious thyroiditis.

4. Graves-Basedow disease.

133- An 83-year-old woman has been admitted for 20 days due to complicated acute diverticulitis. As part of her treatment, she required a sigmoidectomy and has been receiving intravenous antibiotic therapy with ertapenem for 8 days. In recent days she has diarrhea without pathological products, consumes 25% of the recommended intake, and has lost 9 kg since admission; she currently weighs 75 kg and is 1.55 m tall, BMI 31.2 kg/m2. Regarding her nutritional status, what is the most likely diagnosis?:

1. No change in nutritional status.

2. Severe malnutrition related to acute illness.

3. Intestinal failure due to short bowel syndrome.

4. Grade II obesity.

134- A 37-year-old man diagnosed with a 2.6 cm medullary thyroid carcinoma, with no evidence of lymph node involvement in the preoperative study, treated with total thyroidectomy and central compartment lymphadenectomy; staging T2N0M0, stage I. To correctly approach the disease, multiple aspects must be considered. Select the correct answer:

1. Medullary thyroid carcinoma is the second most common histological type of thyroid cancer and can be found in the context of multiple endocrine neoplasia type 2.

2. In most cases, it is sporadic and for biochemical monitoring of the disease, calcitonin, CEA, or thyroglobulin can be used.

3. Replacement therapy with levothyroxine should be adjusted to achieve suppressed TSH.

4. Genetic testing of the RET proto-oncogene is always indicated, as it can be mutated in both hereditary and sporadic medullary carcinomas.

135- In the perioperative management of glycemic control in a diabetic patient undergoing hip arthroplasty, indicate the INCORRECT statement:

1. Intensive glycemic control in the perioperative period often results in improved outcomes.

2. Desired glucose levels will range between 100 and 180 mg/100mL in the perioperative period.

3. If glycemic control is very deficient (HbA1c >9 %), optimization should be attempted before surgery.

4. Optimization of perioperative glycemic control reduces infections and, in critical surgical patients, can reduce mortality.

136- An 80-year-old male from a nursing home presents with urinary sepsis that has not responded to empirical treatment with ceftriaxone. Blood and urine cultures show growth of E. coli resistant to cephalosporins. The laboratory informs us that it is a strain that produces extended-spectrum beta-lactamases. Which of the following antibiotics should be used?:

1. Ertapenem.

2. Amoxicillin/clavulanic.

3. Tigecycline.

4. Ceftazidime.

137- A male consults after having had unprotected sexual intercourse 48 hours ago, with ejaculation, with an HIV-positive person who has been on antiretroviral treatment and has had an undetectable viral load for years. The HIV test performed on the patient at that time in the emergency room is negative. What is the best recommendation we should give him?:

1. He should do nothing as the risk of HIV transmission is negligible.

2. IV gammaglobulin should be recommended within the first 72 hours.

3. He should take post-exposure prophylaxis for HIV for 28 days.

4. He could have taken post-exposure prophylaxis, but it is too late to start now.

138- A 22-year-old patient presents with symptoms of proctitis, including mucus, rectal tenesmus, and fever. On examination, he is found to be febrile and presents bilateral inguinal lymphadenopathies. PCR of the rectal exudate shows Chlamydia trachomatis serovar lymphogranuloma venereum. Which of the following is the best treatment?:

1. Doxycycline 100 mg every 12 hours for 21 days.

2. Cloxacillin 500 mg every 6 hours for 7 days.

3. Ceftriaxone 0.5 g IM (single dose) and azithromycin 1 g orally (single dose).

4. Moxifloxacin 400 mg once a day for 7 days.

139- A 34-year-old male diagnosed with otitis media 5 days ago, with no other medical history, begins with a fever of 39°C, headache, and neck stiffness. A lumbar puncture is performed suggestive of bacterial meningitis. He decides to start empirical treatment until culture and antibiogram results are received. Which of the following would be the best treatment?:

1. Ceftriaxone plus vancomycin.

2. Cefotaxime plus ampicillin.

3. Moxifloxacin plus vancomycin.

4. Meropenem plus ampicillin.

140- Regarding pharyngitis caused by group A Streptococcus pyogenes, it is true that:

1. The clinical picture is diagnostic: high fever, general malaise, sore throat, and occasionally, lymphadenopathy.

2. Rapid diagnostic tests have high sensitivity, so if the result is positive, we can establish a definitive diagnosis.

3. The preferred treatment regimen is amoxicillin-clavulanate 875 mg every 8 hours for 10 days.

4. Antibiotic treatment has been shown to reduce the possibility of rheumatic fever but not post-streptococcal glomerulonephritis.

141- A 72-year-old patient, with a history of diabetes mellitus and hypertension, presents with a three-week history of fever and general malaise, with no apparent source although a cardiac murmur was suspected. Blood cultures were positive for methicillin-resistant Staphylococcus aureus with intermediate sensitivity to vancomycin. An echocardiogram showed an image consistent with vegetation on the mitral valve. He was started on daptomycin 10 mg/kg IV once a day. After seven days of treatment, he continues with fever and presents orthopnea. On examination, there is a 3/6 systolic murmur at the apex, radiating to the left axilla. Chest X-ray shows a bilateral alveolar-interstitial hiliofugal pulmonary infiltrate. Which of the following is the most appropriate course of action?:

1. Indicate urgent valve replacement surgery.

2. Add IV ceftaroline, diuretics, and monitor in intensive care.

3. Discontinue daptomycin and prescribe high-dose vancomycin and gentamicin, along with diuretics and monitoring in intensive care.

4. Request a new echocardiogram, administer diuretic treatment, and monitor in intensive care.

142- A 67-year-old woman who suffered from a Clostridioides difficile infection two months before the consultation. She comes in with symptoms compatible with a recurrent infection by the same germ. What is the most effective treatment to prevent further recurrences of the infection?:

1. Metronidazole.

2. Fidaxomicin.

3. Vancomycin.

4. Rifaximin.

Here's the translation:

143- A 52-year-old woman with difficult-to-control high blood pressure who consults for the appearance of erythematous lesions on the cheeks, chest, and arms, along with polyarthralgia, after starting treatment with hydralazine. Basic autoimmunity tests detect positive antinuclear antibodies of 1:160 (N <1:80). Given the suspicion of drug-induced lupus, which of the following autoantibodies should be investigated?:

1. Anti-histone antibodies.

2. Antineutrophil cytoplasmic antibodies.

3. Antichromatin antibodies.

4. Antiphospholipid antibodies.

144- In a patient recently diagnosed with diffuse scleroderma, what checks should be carried out periodically during the first few months of progression?:

1. Doppler echocardiography and electrocardiogram.

2. Rodnan score (skin thickness test) and capillaroscopy.

3. Kidney function and blood pressure.

4. Respiratory functional tests and chest CT.

145- A 23-year-old Caucasian man, originally from Zaragoza, with no significant medical or family history. Since the age of five, he has had self-limiting episodes of fever up to 39 ºC, diffuse abdominal pain, widespread myalgia, and erythematous lesions on the lower part of his legs. At the age of 10, he had his appendix removed, although no inflammatory signs were evident in the appendix. Episodes last about 2-3 days, improve with NSAIDs, and recur every 1 or 2 months. During febrile outbreaks, physical examination shows diffuse abdominal pain with guarding upon superficial palpation and erysipelas-like lesions on the anterolateral and distal face of the legs, and analysis shows a rise in C-reactive protein up

to 6 mg/dL (N <1 mg/dL), which normalize in periods without fever. What is the most likely initial diagnosis?:

1. Muckle-Wells syndrome (cryopyrin-associated periodic syndrome).

2. Familial Mediterranean fever.

3. Blau syndrome (arthrocutaneous granulomatosis).

4. Periodic syndrome associated with the tumor necrosis factor receptor.

146- A 41-year-old woman with a history of growth delay during childhood without alteration in development stages. She reports exercise intolerance from her youth, which she describes as a lack of skill for sports. In the last 4 years, she mentions an increase in abdominal circumference; an ultrasound showed mild, uniform hepatomegaly without sonographic evidence of cirrhosis. After a mild COVID-19 infection 8 months ago, she develops instability and progressive, symmetrical weakness that prevents her from performing daily life activities, with episodes of sweating. Physical examination: globally decreased muscle strength symmetrically. Mild, non-painful hepatomegaly. Analysis: arterial pH 7.38, PaO_2 99 mmHg, $PaCO_2$ 39 mmHg, blood sugar 49 mg/dL, ASAT/ALAT 63/71 U/L, lactate 14 mg/dL (N: 5-20), ammonia 25 µmol/L (N: 9-33), TSH: 4.6 mUI/L (N: 0.37-4.7), creatine kinase 500 U/L (N: <300), ketonuria ++. An echocardiogram showed slight hypertrophy and dilation of the left ventricle. Which of the following is the most likely diagnosis?:

1. Glycogen storage disease type III.

2. Post COVID-19 syndrome.

3. Primary hyperthyroidism.

4. Beta-oxidation disorder of fatty acids.

147- A 42-year-old woman who for 4 years, with the cold, presents a change in color of the fingers with intense pallor. For a few months, she needs treatment with omeprazole due to occasional retrosternal burning sensation, and the examination shows difficulty pinching the skin on the back of the hands. Which of the following autoantibodies is considered essential for her correct follow-up, due to the prognostic consequences that its positivity would entail?:

1. Anti-DNA.

2. Anti-proteinase 3.

3. Anti-glomerular basement membrane.

4. Anti-RNA polymerase III.

148- At the time of diagnosis of Sjögren's syndrome, which factors are associated with the possibility of developing a B-cell lymphoma?:

1. Fever and lymphadenopathy.

2. Anti-La and anti-Ro antibodies.

3. Splenomegaly and pancytopenia.

4. Hypocomplementemia and cryoglobulin.

149- A 45-year-old male, non-smoker, and does not consume alcohol, presents with progressive shortness of breath over two months. Physical examination: afebrile, respiratory rate 19 rpm, heart rate 120 bpm, SatO2 93%. Skin lesions that appear ulcerative on palms and fingers; crackling rales in both lung bases; normal muscle strength. Lab tests: CK 300 UI/L (N: <150) and aldolase 12 UI/L (N: <6). Chest X-ray: bibasal interstitial pattern. A muscle biopsy showed necrosis of muscle cells, perifascicular atrophy, and perivascular inflammatory infiltrates. Which of the following is the most likely diagnosis?:

1. Dermatomyositis associated with the anti-TIF1-gamma autoantibody (transcription intermediary factor 1-gamma).

2. Idiopathic inflammatory myopathy associated with the anti-FHL-1 autoantibody (Four and a half LIM domains 1).

3. Dermatomyositis associated with the anti-MDA-5 autoantibody (melanoma differentiation-associated gene 5).

4. Myofibrillar myopathy associated with the anti-desmin antibody.

150- A 56-year-old male comes in for a type 2 diabetes mellitus check-up. He's been on metformin 850 mg twice a day for eight weeks. He reports feeling well, with no excessive thirst or urination, and blood sugar levels between 140-180 mg/dL. His latest HbA1c was 7.7%. Physical examination: blood pressure 137/86 mmHg and pulse 74 bpm. Fundus examination reveals mild proliferative retinopathy. Urine sediment shows microalbuminuria of 180 mg/g creatinine. What is the next most appropriate step?:

1. 24-hour urine test for protein and creatinine.

2. Indicate a renal ultrasound.

3. Start treatment with calcium channel blockers.

4. Start treatment with ACE inhibitors.

151- A 65-year-old woman presents with hypercholesterolemia. She denies any personal or family history of dyslipidemia. Lab tests show: total cholesterol 500 mg/dL, LDL cholesterol 350 mg/dL, HDL 38 mg/dL, and triglycerides 206 mg/dL. The rest of the analysis and thyroid hormones are normal, except total proteins 5.9 g/dL (N: 6.4-8.3) and albumin 3 g/dL (N: 3.5-5). Basic urine test with an albumin/creatinine ratio of 2,000 mg/g and proteinuria +++. Physical examination shows no xanthomas or corneal arcus, but mild ankle edema is present. Which of the following is the most likely diagnosis?:

1. Dyslipidemia secondary to nephrotic syndrome.

2. Heterozygous familial hypercholesterolemia.

3. Homozygous familial hypercholesterolemia.

4. Recessive familial hypercholesterolemia due to LDLRAP1 gene mutation.

152- Which type of patient wishes CANNOT be addressed in an advance care planning (or shared care planning)?:

1. Rejection of life-threatening interventions.

2. Organ donation.

3. Rejection of non-life-threatening interventions.

4. Implementation of futile interventions, with no clinical indication.

153- An 89-year-old woman, institutionalized in a nursing home for 5 years due to an 8-year Alzheimer's diagnosis. She's dependent on all basic daily activities and has severe cognitive impairment. She doesn't recognize family and cannot produce language. She's admitted for a respiratory infection due to aspiration. After completing an antibiotic cycle, she recovers well, but swallowing issues with a high risk of aspiration persist. The doctor suggests to the children not to start enteral feeding via tubes, but rather let the disease progress naturally, only treating potential complications symptomatically. What ethical concept underlies this clinical decision?:

1. Treatment rejection.

2. Active euthanasia.

3. Therapeutic effort limitation.

4. Therapeutic obstinacy.

154- A 48-year-old patient, diagnosed with amyotrophic lateral sclerosis 3 years ago, requires help with all basic daily activities and uses a tube for feeding. In the past year, he's been hospitalized multiple times for respiratory infections. Currently, he's admitted for another respiratory infection with associated global respiratory failure. He requires non-invasive mechanical ventilation as part of his treatment. After 5 days in the hospital, without clinical improvement, he expresses unbearable suffering, finds no reason to continue living this way, and asks for the removal of the mechanical ventilation, even if it causes his death. After several conversations, you confirm with the patient that this is his wish, and his family supports the decision. This is a decision of:

1. Treatment rejection.

2. Limitation of therapeutic effort.

3. Therapeutic obstinacy.

4. Euthanasia.

155- A 65-year-old male is brought to the emergency department intubated and sedated due to polytrauma following a traffic accident. After the initial assessment, a splenic laceration is found requiring urgent surgical intervention. How should the consent process and the surgery consent form signature proceed?:

1. An urgent surgical intervention is not possible when only verbal consent is obtained from a patient's representative.

2. If contact with any family or close friend of the patient is not possible, the surgical intervention cannot be performed.

3. The attending physician should try to contact the patient's family, explain the situation, and obtain their consent for the procedure.

4. Without a signed informed consent form from the patient's family, the intervention cannot be performed.

156- Regarding the treatment of depression with antidepressant drugs in terminally ill patients. Which of the following is the best option?:

1. The initial dose of selective serotonin reuptake inhibitors should be the same as that used in adults without terminal illness.

2. Tricyclic antidepressants are recommended to be avoided unless used as adjuncts in pain treatment.

3. In patients where symptoms like anxiety or insomnia dominate, fluoxetine is preferred.

4. Trazodone, even at low doses, is contraindicated due to its frequent side effects.

157- Regarding the treatment of constipation in terminally ill patients, identify the INCORRECT response:

1. In the presence of opioid treatment, vegetable fiber supplements are recommended.

2. The fundamental elements of treatment are stimulant and osmotic laxatives, stool softeners, fluids, and enemas.

3. To prevent constipation caused by opioids, a combination of a laxative and a stool softener should be used.

4. If there's no bowel movement after several days of treatment, a rectal exam is needed to remove impacted material.

158- A 95-year-old male is admitted to the emergency department of the hospital with a history of cranial trauma due to an accidental fall at home. On a cranial CT scan, an acute epidural hematoma is observed. He passes away shortly after admission in the emergency department itself. From a legal standpoint, the responsible physician is obliged to complete only one of the following documents:

1. Regular death certificate.

2. Statistical death bulletin with judicial intervention (violent death).

3. Statistical death bulletin.

4. Injury report.

159- If a person under 18 years of age wishes to be a living organ donor. Which of the following statements is true?:

1. If the person is between 16 and 18 years old, the decision belongs to the minor if they prove they understand the extent of the procedure. However, parents or guardians must be consulted.

2. If the person is between 16 and 18 years old, the decision belongs to the parents or legal guardians, but the minor must be consulted if they prove they understand the extent of the procedure.

3. In case of conflict between the minor's and the parents' or guardians' criteria, a judge should decide applying the principle of the minor's best interest.

4. Minors under 18 years of age cannot be living organ donors, according to the Law. There are no exceptions.

160- Regarding scabies, identify the INCORRECT statement:

1. It is a highly contagious skin infestation caused by Sarcoptes scabiei.

2. It's a polymorphic eruption where lesions caused by the mite itself are found in the interdigital folds and wrists, and to a lesser extent in the armpits, breasts, and penis.

3. In very persistent adult cases, lesions tend to generalize across the trunk and face.

4. Dermatoscopic analysis of the end of a burrow may reveal the mite, which appears as a delta wing.

161- A 49-year-old woman, who frequently visits the doctor, comes to the consultation claiming she needs sick leave, having not gone to work for several days due to dizziness. After a complete examination and detailed anamnesis, you detect that she is a victim of violence from her partner, a situation she had never mentioned before. At what level of prevention do you place the doctor's action?:

1. Primary prevention.

2. Secondary prevention.

3. Tertiary prevention.

4. Quaternary prevention.

162- A 50-year-old man with no significant history other than smoking 20 cigarettes a day, leads a sedentary life, and takes no chronic medication. Upon examination: abdominal circumference 104 cm, blood pressure 136/89 mmHg. Laboratory results include: triglycerides 160 mg/dL, HDL cholesterol 30 mg/dL, and fasting glucose 88 mg/dL. According to the diagnostic criteria for metabolic syndrome of the ATPIII, 2001 (Adult Treatment Panel III. The National Cholesterol Education Program). How many criteria does he meet?:

1. Two criteria.

2. Three criteria.

3. Four criteria.

4. Five criteria.

163- A 50-year-old patient consults for a localized lymph node enlargement. Which of the following locations suggests a higher risk of malignancy?:

1. Retroauricular.

2. Supraclavicular.

3. Axillary.

4. Inguinal.

164- A 70-year-old man with a history of hypertension and dyslipidemia consults his family doctor after a recent admission for heart failure. He is currently asymptomatic, treated with enalapril, furosemide, atorvastatin, and acetylsalicylic acid. Physical examination reveals a heart rate of 78 bpm, with no signs of heart failure. The echocardiogram performed during admission shows left ventricular hypertrophy and an ejection fraction of 30%. What is the most appropriate therapeutic approach?:

1. Add bisoprolol.

2. Add diltiazem.

3. Discontinue enalapril and start losartan.

4. No changes in medication.

165- A 50-year-old patient with diabetes mellitus being treated with insulin. What is the recommended target for postprandial capillary plasma glucose concentration?:

1. < 126 mg/dL.

2. < 140 mg/dL.

3. < 180 mg/dL.

4. < 220 mg/dL.

166- In patients with type 2 diabetes mellitus on long-term treatment with the same drug, which of the following is NOT associated with a reduced likelihood of cardiovascular complications?:

1. Sodium-glucose cotransporter 2 inhibitors (SGLT2i).

2. Glucagon-like peptide 1 receptor agonists (GLP-1 RA).

3. Insulin.

4. Metformin.

167- A 14-year-old girl is taken to the emergency department by her parents after having ingested multiple paracetamol tablets 12 hours prior (they are unsure of the exact amount). She is nauseous, pale, and sweaty. Paracetamol levels in the blood are found in the range of probable liver toxicity. Liver function tests and coagulation are normal. What is the next course of action?:

1. Observe and repeat paracetamol levels in 4 hours.

2. Start treatment with N-acetylcysteine and maintain for 12 hours.

3. Observe and repeat paracetamol levels, liver tests, and coagulation in 4 hours.

4. Start treatment with N-acetylcysteine and maintain based on the evolution of paracetamol levels, liver, and renal tests.

168- In the initial management of a patient with septic shock in the emergency room, indicate the INCORRECT statement:

1. Serum lactate should be determined as a marker of tissue hypoperfusion.

2. Early (1st hour) sample collection for cultures and initiation of broad-spectrum antibiotic therapy should be carried out.

3. Volume replacement should be performed with crystalloids within the first 3 hours.

4. Dopamine is recommended as the first-choice vasopressor.

169- A 70-year-old homeless man, exposed to high temperatures for hours, is taken to the emergency room on suspicion of heatstroke. He presents with a temperature of 40.5°C, disorientation, and decreased consciousness. Which of the following is the most correct initial action?:

1. General support measures and immersion bath to reduce body temperature.

2. Support measures, cooling with wet sponges (15-20°C) on the body surface, and ensuring adequate intravenous hydration.

3. The use of cooling blankets is the most effective measure for rapid reduction of body temperature.

4. Support measures, cooling with sponges soaked in alcohol applied to the body surface, and antipyretics.

170- The quick SOFA or qSOFA (Quick Sequential Organ Failure Assessment) is a prognostic index in patients with infection. If a patient comes to the emergency room of a health center with an infectious condition and on examination has 15 points on the Glasgow scale, a heart rate of 110 bpm, a respiratory rate of 18 rpm, a temperature of 38°C, and a systolic blood pressure of 92 mmHg, what score would they get using the qSOFA?:

1. One point.

2. Two points.

3. Three points.

4. Four points.

171- A 65-year-old woman is rescued from a fire in a plastic container warehouse and taken to the emergency room with a headache, nausea, vomiting, and drowsiness after smoke inhalation. Of the following, which is the most suspected diagnosis and the most correct initial treatment?:

1. Chlorine poisoning. Bicarbonate nebulization (4 ml of 0.9% saline + 6 mL of 1M sodium bicarbonate) in the first 3 hours after exposure.

2. Inhalation of nitrous vapors. Symptomatic and supportive treatment.

3. Carbon monoxide and cyanide poisoning. Supportive measures along with 100% oxygen therapy and hydroxocobalamin at a dose of 5 g IV in 10 minutes.

4. Toxic skin absorption poisoning. Supportive measures, oxygen therapy, skin cleaning, and atropine (1 mg IV) which can be repeated every 2-3 minutes until clinical improvement.

172- A 59-year-old man with no medical history presents with asthenia and exertional dyspnea of several months' duration. His medical record shows that he hasn't visited the doctor in more than ten years. Physical examination detects conjunctival pallor, with the rest being normal. The electrocardiogram is normal, and the blood count highlights: Hb 10.6 g/dL, Hct 31%, MCV 75 fl, red cell distribution width 22%. Which of the following would be the appropriate initial step?:

1. Rectal examination and schedule colonoscopy.

2. Spirometry and schedule bronchoscopy.

3. Measure haptoglobin and perform Coombs test.

4. Measure vitamin B12 and schedule bone marrow biopsy.

173- Cancer patients can present with cardiovascular complications such as superior vena cava syndrome. Indicate in which of the following neoplasms this complication is most common:

1. Lymphoma.

2. Breast cancer.

3. Lung cancer.

4. Mediastinal lymph node metastases from other origins.

174- A 75-year-old man is admitted to the hospital with rapidly progressive renal failure,anuria, poor general condition, and fever. Laboratory data shows positive ANCAs and negative anti-MBG antibodies. A kidney biopsy is performed. Hematoxylin-eosin staining reveals necrotizing glomerulonephritis with extracapillary epithelial reaction and crescent formation in 80% of the glomeruli. Direct immunofluorescence techniques for IgA, IgG, IgM, C3, C1q, kappa, and lambda are negative. What is the histopathological diagnosis?:

1. Postinfectious glomerulonephritis.

2. Pauci-immune vasculitis.

3. IgA nephropathy.

4. Goodpasture's syndrome.

175- A 30-year-old man with a history of upper gastrointestinal bleeding 1 year ago. In the gastroscopy, several peptic ulcers were observed. He consults for a kidney colic episode. The analysis shows calcium 11.1 mg/dL (N: 8.6-10.3 mg/dL), parathyroid hormone 150 ng/L (N: 8-51 ng/L) with normal levels of albumin and 25-OH vitamin D. Which syndrome are these clinical and analytical data related to?:

1. Carney's complex.

2. Polyglandular syndrome type 1.

3. Multiple endocrine neoplasia type 2A.

4. Multiple endocrine neoplasia type 1.

176- The tumor lysis syndrome, common in hematological neoplasms, is characterized by the following complications, EXCEPT:

1. Hypercalcemia.

2. Hyperuricemia.

3. Hyperphosphatemia.

4. Uremia.

Sure, here's the translation:

177- A 35-year-old male has been experiencing pain and inflammation in the 2nd and 3rd metacarpophalangeal joints of the right hand and in the 3rd and 4th interphalangeal joints of the left hand for 3 months. Upon physical examination, flaky skin lesions are observed on the extensor areas of the elbows and on the right knee. Initial tests show ESR 34 mm, CRP 1.8 mg/dL (N: 0-0.5), negative rheumatoid factor, and negative anti-cyclic citrullinated peptide antibodies. Hand X-rays: soft tissue swelling without erosions. Which of the following diagnoses is most likely?:

1. Rheumatoid arthritis.

2. Systemic lupus erythematosus.

3. Psoriatic arthritis.

4. Osteoarthritis.

178- A 20-year-old female presents with raised violet lesions on the distal area of her toes during winter months. She mentions itching and occasional appearance of wounds on her fingers that heal within a few days. Which of the following is the most likely diagnosis?:

1. Raynaud's phenomenon.

2. Chilblains (Erythema pernio).

3. Acrocyanosis.

4. Erythromelalgia.

179- Regarding thoracic diseases related to asbestos exposure, identify the INCORRECT answer:

1. The presence of pleural effusion in a patient with known asbestos exposure indicates malignant mesothelioma.

2. Irregular linear opacities at the lung bases are the characteristic radiographic signs of asbestosis.

3. Lung cancer is the most common tumor associated with asbestos exposure.

4. Pleural plaques indicate only exposure but not lung involvement.

180- A 68-year-old male presents with a 4-day history of generalized malaise, fever, and cough with expectoration. Physical examination: bibasal crepitations. Chest X-ray: infiltrates with a peripheral alveolo-interstitial pattern. Positive PCR for SARS-CoV 2. The ratio of partial pressure of oxygen in arterial blood to inspired oxygen fraction is 250. Which of the following is the most likely diagnosis?:

1. Pulmonary hemorrhage secondary to SARS-CoV 2 infection.

2. Bacterial pneumonia in a SARS-CoV2 positive patient.

3. SARS-CoV 2 pneumonia with acute respiratory distress syndrome.

4. Severe SARS-CoV 2 infection with alveolar hypoventilation.

181- A 56-year-old male with a hiatal hernia and persistent rhinitis due to sensitivity to Arizona cypress trees consults due to worsening symptoms over the last few weeks. These include nasal

blockage, tearing, cough, exertional dyspnea, and inspiratory wheezing, which don't improve with his usual bronchodilator and inhaled corticosteroid. He has lived with a dog for 5 years. He has used a humidifier for the past 3 months at night due to a dry throat. Lung auscultation: fine crackles at bases. Spirometry: FVC 2740 (68%), FEV1 2530 (79%), FEV1/FVC 0.92. Chest CT: subpleural infiltrates with a fine reticular pattern in both lower lobes and some patchy ones in the upper lobes. What is the most likely diagnosis and recommended action?:

1. Hypersensitivity pneumonitis. It's recommended to remove the humidifier and monitor progress.

2. Cough related to gastroesophageal reflux. It's recommended to start proton pump inhibitors.

3. Asthma crisis in a patient sensitized to pollen. It's recommended to add systemic corticosteroids.

4. Animal hair allergy. Allergy testing is recommended.

182- A 62-year-old patient consults six months after suffering an acute myocardial infarction. He currently smokes 15 cigarettes a day, is overweight, and leads a sedentary lifestyle. He is on treatment with atorvastatin 80 mg/day and ramipril 5 mg/day. His tests show LDL cholesterol 68 mg/dL, blood glucose 115 mg/dL, Hb1Ac 5.7%, no microalbuminuria, and a blood pressure of 140/85 mmHg during the consultation. What's the most effective measure from the following to prevent another cardiovascular event?:

1. Optimize the treatment of high blood pressure as it is poorly controlled.

2. Optimize lipid-lowering treatment as it is poorly controlled.

3. Add a drug for diabetes.

4. Quit smoking, walk daily, and lose weight.
