# Supplementary material for: Evaluating the Efficacy of ChatGPT in Navigating the Spanish Medical Residency Entrance Examination (MIR): Promising Horizons for AI in Clinical Medicine
Source: Clin Pract. 2023 Nov 20;13(6):1460–87. doi: 10.3390/clinpract13060130 (PMC10660543; doi:10.3390/clinpract13060130)
Supplement: Supplementary file 1 [file clinpract-13-00130-s001.zip › Table S2 Answer to MIR examination questions.pdf]

Table S2. Answer to MIR examination questions.

QN	CA	QN	CA	QN	CA	v	RC	v	RC
1	2	38	3	75	2	112	2	149	3
2	2	39	2	76	4	113	4	150	4
3	3	40	3	77	4	114	1	151	1
4	3	41	2	78	2	115	3	152	4
5	1	42	1	79	1	116	3	153	3
6	3	43	4	80	3	117	2	154	1
7	1	44	4	81	4	118	1	155	3
8	2	45	2	82	3	119	3	156	2
9	1	46	4	83	2	120	1	157	1
10	2	47	1	84	1	121	3	158	4
11	4	48	2	85	3	122	2	159	4
12	3	49	4	86	1	123	1	160	3
13	4	50	2	87	2	124	2	161	2
14	3	51	3	88	4	125	3	162	3
15	1	52	3	89	1	126	4	163	2
16	2	53	2	90	1	127	2	164	1
17	1	54	1	91	1	128	3	165	3
18	2	55	1	92	4	129	1	166	3
19	1	56	3	93	2	130	2	167	4
20	1	57	4	94	2	131	3	168	4
21	4	58	1	95	3	132	1	169	2
22	2	59	1	96	1	133	2	170	1
23	4	60	3	97	4	134	4	171	3
24	4	61	4	98	2	135	1	172	1
25	4	62	3	99	4	136	1	173	3
26	3	63	4	100	3	137	1	174	2
27	2	64	4	101	4	138	1	175	4
28	2	65	1	102	4	139	1	176	1
29	3	66	2	103	3	140	4	177	3
30	1	67	1	104	2	141	1	178	2
31	4	68	4	105	3	142	2	179	1
32	3	69	3	106	4	143	1	180	3
33	2	70	3	107	3	144	3	181	1
34	3	71	2	108	2	145	2	182	4
35	2	72	1	109	2	146	1		
36	4	73	2	110	3	147	4		
37	3	74	4	111	2	148	4		

QN= Question Number CA= Correct Answer
