# Supplementary material for: Evaluating the Efficacy of ChatGPT in Navigating the Spanish Medical Residency Entrance Examination (MIR): Promising Horizons for AI in Clinical Medicine
Source: Clin Pract. 2023 Nov 20;13(6):1460–87. doi: 10.3390/clinpract13060130 (PMC10660543; doi:10.3390/clinpract13060130)
Supplement: Supplementary file 1 [file clinpract-13-00130-s001.zip › Table S3 Questions failed by GPT4 indicating the wrong answer and the classification of the Error.pdf]

Table S3 Questions failed by GPT4 in the MIR examination, indicating the correct answer, the wrong answer, and the Error classification.

Question	Correct Answer	GPT4 Answer	Error	Category Error classification
20- The reason an intervention has a "strong" recommendation grade in a clinical practice guideline is:	It has more benefits than risks.	It's based on a high level of evidence.	A.	A. Circumstances or events that have the capacity to cause error.
21- In which of the following diseases should we apply contact and air (aerosol) isolation precautions?	Chickenpox	Measles.	B	B. Error did not reach the patient.
22- In the forest plot that displays the results of a systematic review of studies, the square next to each study represents	The estimator of interest (like relative risk) for each study.	The weight of each study when conducting a meta-analysis of said studies	A.	A. Circumstances or events that have the capacity to cause error.
27- In managing a pregnant woman diagnosed with bipolar disorder, which of the following drugs is effective in this disease and has been established as safer in pregnancy?:	Olanzapine.	Lithium.	D	D. Error reached the patient and required monitoring
30- In a patient with a duodenal ulcer and a high cardiovascular risk due to ischemic heart disease, if they require treatment with an NSAID, which of the following would you recommend as safer?:	Naproxen.	Celecoxib.	C	C. An error occurred that reached the patient but did not cause the patient harm.
31- A 68-year-old man with a history of obesity and type 2 diabetes mellitus, treated with metformin, is admitted for a first episode of heart failure. He has a glycosylated hemoglobin of 8.5%. To optimize diabetes treatment upon discharge, which of the following drugs is contraindicated?:	Pioglitazone.	Canagliflozin	E	E. An error that may have contributed to or resulted in temporary harm to the patient and required intervention.
44- A 35-year-old primigravida in the second stage of labor with epidural analgesia. When would the application of the vacuum to shorten the expulsive period be indicated?:	When fetal bradycardia occurs that doesn't recover after contraction ends, and the posterior fontanel is past the third Hodge plane.	When, after 2 hours in full dilation, the baby's position remains at the second Hodge plane with maternal pushing.	D	D. Error reached the patient and required monitoring
57- For the diagnosis of classic Kawasaki disease, the presence of fever for at least five days and 4 of the main symptoms of the disease are required. Of the following, indicate which is NOT CONSIDERED a main sign:	Bilateral exudative conjunctival injection.	Polymorphic rash.	A	A. Circumstances or events that have the capacity to cause error.
78- Regarding post-traumatic epileptic seizures, it's true that:	Penetrating injuries and the severity of the injury are favoring factors.	They are common immediately after the impact.	A	A. Circumstances or events that have the capacity to cause error.
80- A 50-year-old man with Legionella pneumonia requires ICU admission due to acute respiratory failure. He needs to be intubated and connected to invasive mechanical ventilation. He's diagnosed with acute respiratory distress syndrome and is placed in the prone position. Regarding this positional technique, indicate the INCORRECT statement:	The ratio between the partial pressure of oxygen and the fraction of inspired oxygen below which it is recommended is 300 mmHg.	The prone position during invasive mechanical ventilation requires deep sedation	F	F. An error occurred that may have contributed to or resulted in temporary harm to the patient and required initial or prolonged hospitalization
81- A 58-year-old patient suffers from COVID-19 pneumonia and requires intubation with mechanical ventilation in the ICU. A week later, a tracheostomy is performed, but the patient still requires mechanical ventilation. A motor deficit is detected in all four limbs with generalized hyporeflexia. The initial diagnostic orientation and therapeutic attitude to follow are:	Critical illness myopathy. Intensive respiratory and motor rehabilitation	Guillain-Barré Syndrome. Confirm the diagnosis with complementary tests.	F	F. An error occurred that may have contributed to or resulted in temporary harm to the patient and required initial or prolonged hospitalization
98- A 75-year-old male reports progressive dyspnea, with decreasing exercise tolerance. He reports syncope on two occasions in the past few months, coinciding with moderate effort, and occasionally reports chest pain that increases with effort. On examination, the presence of a parvus et tardus arterial pulse and a mesosystolic murmur stand out. The blood test is normal except for a creatinine of 2.2 mg/dL. Surgical risk assessment establishes an intermediate risk. Indicate which of the following therapeutic options is most appropriate	Percutaneous implantation of the aortic valve (TAVI).	Surgical replacement of the aortic valve.	G	G. An error occurred that may have contributed to or resulted in permanent patient harm

Question	Right Answer	GPT4 Answer	Error	Category Error classification
99- A 43-year-old woman, a professional swimmer, with no relevant medical history, reports swelling and heaviness of her right arm, lasting 72 hours. On examination, there is edema of the right upper limb with the presence of dilated veins in the pectoral region. Regarding the diagnostic approach, indicate the INCORRECT answer:	A phlebography should be performed to confirm the presence of axillo-subclavian venous thrombosis.	A simple chest x-ray should be performed to detect bone abnormalities.	E	E. An error that may have contributed to or resulted in temporary harm to the patient and required intervention.
101- A 67-year-old male diagnosed with COPD with a GOLD 1 category, smoker, reports dyspnea with intense efforts, without exacerbations. Which of the following is the most correct therapeutic recommendation?	Non-pharmacological treatment	Regular treatment with long-acting muscarinic bronchodilators (LAMA).	C	C. An error occurred that reached the patient but did not cause the patient harm.
102- A 70-year-old woman admitted for severe traumatic brain injury with secondary subarachnoid hemorrhage. On the second day of admission, she experiences dyspnea and chest pain. Chest angio CT confirms the diagnosis of pulmonary embolism. The treatment of choice is:	Inferior vena cava filter.	Low molecular weight heparin.	D	D. An error occurred that reached the patient and required monitoring to confirm that it resulted in no harm to the patient or required intervention to preclude harm.
112- A 45-year-old man is brought to the emergency department after having tonic-clonic seizures in public. He is confused and gives incoherent and vague answers. Reviewing his history, he is diagnosed with schizophrenia and depression, but he seems to have been off his medication for weeks. Examination: Stuporous, with no other findings. Blood tests with normal hemogram, glucose 98 mg/dL, urea 37 mg/dL, creatinine 0.8 mg/dL, Na 118 mEq/L, K 3.4 mEq/L, Ca 9.7 mg/dL, serum osmolarity 252 mOsm/kg, urinary osmolarity 78 mOsm/kg, urine density 1002, normal sediment. What is the most likely cause of his electrolyte disturbances?	Primary polydipsia.	Syndrome of inappropriate ADH secretion	A	A. Circumstances or events that have the capacity to cause error.
137- A male consults after having had unprotected sexual intercourse 48 hours ago, with ejaculation, with an HIV-positive person who has been on antiretroviral treatment and has had an undetectable viral load for years. The HIV test performed on the patient at that time in the emergency room is negative. What is the best recommendation we should give him?:	He should do nothing as the risk of HIV transmission is negligible.	He should take post-exposure prophylaxis for HIV for 28 days	C	C. An error occurred that reached the patient but did not cause the patient harm.
140- Regarding pharyngitis caused by group A Streptococcus pyogenes, it is true that:	Antibiotic treatment has been shown to reduce the possibility of rheumatic fever but not post-streptococcal glomerulonephritis.	The clinical picture is diagnostic: high fever, general malaise, sore throat, and occasionally, lymphadenopathy.	D	D. An error occurred that reached the patient and required monitoring to confirm that it resulted in no harm to the patient or required intervention to preclude harm.
141- A 72-year-old patient, with a history of diabetes mellitus and hypertension, presents with a three-week history of fever and general malaise, with no apparent source although a cardiac murmur was suspected. Blood cultures were positive for methicillin-resistant Staphylococcus aureus with intermediate sensitivity to vancomycin. An echocardiogram showed an image consistent with vegetation on the mitral valve. He was started on daptomycin 10 mg/kg IV once a day. After seven days of treatment, he continues with fever and presents orthopnea. On examination, there is a 3/6 systolic murmur at the apex, radiating to the left axilla. Chest X-ray shows a bilateral alveolar-interstitial hiliofugal pulmonary infiltrate. Which of the following is the most appropriate course of action	Indicate urgent valve replacement surgery.	Add IV ceftaroline, diuretics, and monitor in intensive care.	G	G. An error occurred that may have contributed to or resulted in permanent patient harm.
144- In a patient recently diagnosed with diffuse scleroderma, what checks should be carried out periodically during the first few months of progression?:	Kidney function and blood pressure.	Doppler echocardiography and electrocardiogram.	A	A. Circumstances or events that have the capacity to cause error.

Question	Right Answer	GPT4 Answer	Error	Category Error classification
148- At the time of diagnosis of Sjögren's syndrome, which factors are associated with the possibility of developing a B-cell lymphoma?:	Hypocomplementemia and cryoglobulin.	Splenomegaly and pancytopenia.	A	A. Circumstances or events that have the capacity to cause error.
158- A 95-year-old male is admitted to the emergency department of the hospital with a history of cranial trauma due to an accidental fall at home. On a cranial CT scan, an acute epidural hematoma is observed. He passes away shortly after admission in the emergency department itself. From a legal standpoint, the responsible physician is obliged to complete only one of the following documents:	Injury report.	Statistical death bulletin with judicial intervention (violent death).	A	A. Circumstances or events that have the capacity to cause error.
162- A 50-year-old man with no significant history other than smoking 20 cigarettes a day, leads a sedentary life, and takes no chronic medication. Upon examination: abdominal circumference 104 cm, blood pressure 136/89 mmHg. Laboratory results include: triglycerides 160 mg/dL, HDL cholesterol 30 mg/dL, and fasting glucose 88 mg/dL. According to the diagnostic criteria for metabolic syndrome of the ATPIII, 2001 (Adult Treatment Panel III. The National Cholesterol Education Program). How many criteria does he meet?:	Four criteria.	Three criteria.	A	A. Circumstances or events that have the capacity to cause error.
173- Cancer patients can present with cardiovascular complications such as superior vena cava syndrome. Indicate in which of the following neoplasms this complication is most common:	Lung cancer	Lymphoma.	A	A. Circumstances or events that have the capacity to cause error.
